# Carbon-Coated Three-Dimensional MXene/Iron Selenide Ball with Core–Shell Structure for High-Performance Potassium-Ion Batteries

**DOI:** 10.1007/s40820-021-00741-0

**Published:** 2021-12-06

**Authors:** Su Hyun Yang, Yun Jae Lee, Heemin Kang, Seung-Keun Park, Yun Chan Kang

**Affiliations:** 1 Department of Materials Science and Engineering, Korea University, Anam-Dong, Seongbuk-Gu, Seoul, 136-713 Republic of Korea; 2 Department of Advanced Materials Engineering, Chung-Ang University, 4726 Seodong-daero, Daedeok-myeon, Anseong-si, Gyeonggi-do 17546 Republic of Korea

**Keywords:** MXene, Spray pyrolysis, Iron selenide, Potassium-ion batteries, 3D structures

## Abstract

Two-dimensional (2D) MXenes are promising as electrode materials for energy storage, owing to their high electronic conductivity and low diffusion barrier. Unfortunately, similar to most 2D materials, MXene nanosheets easily restack during the electrode preparation, which degrades the electrochemical performance of MXene-based materials. A novel synthetic strategy is proposed for converting MXene into restacking-inhibited three-dimensional (3D) balls coated with iron selenides and carbon. This strategy involves the preparation of Fe_2_O_3_@carbon/MXene microspheres via a facile ultrasonic spray pyrolysis and subsequent selenization process. Such 3D structuring effectively prevents interlayer restacking, increases the surface area, and accelerates ion transport, while maintaining the attractive properties of MXene. Furthermore, combining iron selenides and carbon with 3D MXene balls offers many more sites for ion storage and enhances the structural robustness of the composite balls. The resultant 3D structured microspheres exhibit a high reversible capacity of 410 mAh g^−1^ after 200 cycles at 0.1 A g^−1^ in potassium-ion batteries, corresponding to the capacity retention of 97% as calculated based on 100 cycles. Even at a high current density of 5.0 A g^−1^, the composite exhibits a discharge capacity of 169 mAh g^−1^.

**Supplementary Information:**

The online version contains supplementary material available at 10.1007/s40820-021-00741-0.

## Highlights

- We propose a novel synthetic strategy for converting MXene nanosheets into 3D balls coated with iron selenides and carbon (FeSe_*x*_@C/MB), using the ultrasonic spray pyrolysis and thermal treatment.
- Combining iron selenides and carbon with 3D MXene balls offer many more sites for ion storage and enhance the structural robustness of the composite balls.
- The resultant shows high electrochemical performances when used in potassium-ion battery in terms of cycling stability and rate capability.

**Supplementary Information:**

The online version contains supplementary material available at 10.1007/s40820-021-00741-0.

## Graphical abstract

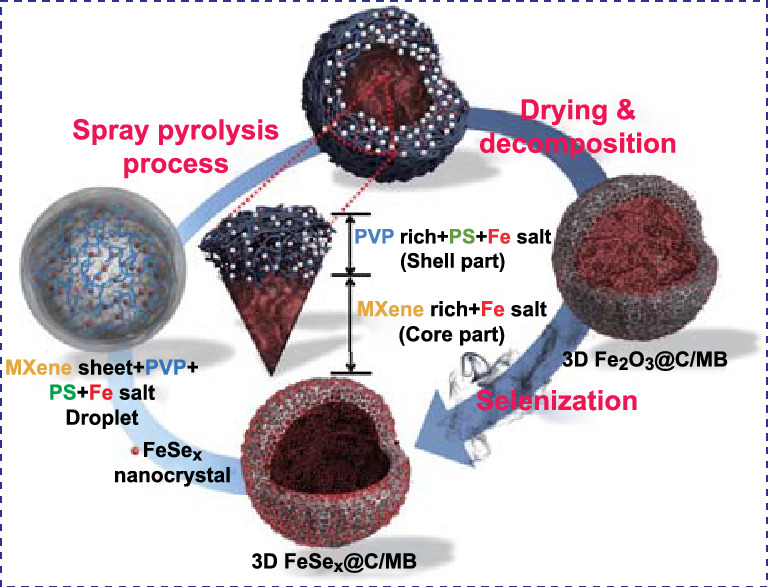

## Introduction

K-ion batteries (KIBs) have garnered significant attention for applications in grid energy storage systems, owing to their high energy density and low cost [1–4]. However, developing suitable electrode materials for high-performance KIBs remains challenging because of the sluggish reaction kinetics and huge volume expansion caused by the larger diameter of K^+^ than those of Li^+^ and Na^+^ [5]. Two-dimensional (2D) materials, including layered transition metal compounds, graphene, and phosphorene, have been considered as promising electrode materials for alkali-ion batteries, owing to their large surface-to-volume ratio and short diffusion pathways [6–10].

As a novel family of 2D materials, MXene has been intensively studied as an anode for metal ion batteries in recent years, because of its high metallic conductivity and low diffusion barrier of ions, which lead to fast electron transport and excellent ion diffusion kinetics [11–14]. In addition, theoretical simulations and the corresponding experimental results demonstrated that its electrochemical properties could be further improved by exfoliating multilayer MXene nanosheets into a few layers [15, 16]. Unfortunately, as is the case with most 2D materials, aggregation and restacking between MXene nanosheets easily occurs during the drying and electrode preparation steps, owing to the van der Waals attraction and H-bonding [17]. These drawbacks can decrease the accessibility of ions and the surface area of electrode materials, degrading their electrochemical performance.

Recently, three-dimensional (3D) constructs by self-assembly of MXene nanosheets have been proposed as one of the effective ways to avoid the restacking problem while retaining the unique properties of MXene [18]. This approach not only inhibits the aggregation/restacking between MXene nanosheets, but also enhances the diffusion of ions, improving the electrochemical performance of the associated electrodes. Correspondingly, several research groups have attempted to develop 3D structured MXene-based electrode materials for high-performance energy storage systems. For example, Gogotsi et al. fabricated 3D MXene hollow spheres for Na-ion storage, using a templated method [17]. The obtained hollow spheres had excellent structural stability and exhibited significantly improved Na-ion storage performance, compared with multilayer MXene. Xu et al. proposed a facile approach for directly transforming MXene nanosheets into 3D carbon-coated MXene architectures [19]. This was achieved by the self-polymerization of dopamine, and the resulting composites exhibited excellent electrochemical performance for Li/Na-ion batteries. Nevertheless, the intrinsically low specific capacity of MXene hinders its applicability. To overcome this problem, it has been proposed to combine MXene with transition metal chalcogenides (TMCs), of which iron selenide is attracting attention as an anode of batteries due to its high theoretical capacity [20–23]; however, facile production of 3D structured MXene/TMC electrode materials without aggregation remains challenging.

Ultrasonic spray pyrolysis is a scalable and cost-effective technique based on the aerosol process, which has been utilized for synthesizing various structured nanomaterials [24–26]. In particular, this method allows to easily transform low-dimensional materials into 3D spheres by a rapid evaporation of droplets. Nevertheless, synthesis of 3D MXene-based electrode materials for high-performance KIBs using the ultrasonic spray pyrolysis approach has been rarely reported until now.

Herein, we propose a novel synthetic strategy for converting MXene nanosheets into 3D balls coated with iron selenides and carbon, using the ultrasonic spray pyrolysis approach, followed by a thermal treatment (denoted as FeSe_*x*_@C/MB). Structuring 2D MXene in 3D could effectively prevent restacking between interlayers, increase surface area, and accelerate ion transport, while maintaining the attractive properties of MXene. Furthermore, combining iron selenides and carbon with 3D MXene balls (denoted as MBs) offers many more sites for ion storage and enhances the structural robustness of the composite balls. The resultant FeSe_*x*_@C/MB anodes exhibit a high reversible capacity, long cycling stability, and excellent rate capability when used in KIBs.

## Experimental Section

3D MXene balls (MBs) decorated with iron selenides and carbon (FeSe_*x*_@C/MB) were synthesized by ultrasonic spray pyrolysis and subsequent thermal treatment for selenization under a reducing atmosphere. MXene sheets were prepared by etching and freeze-drying, as reported previously [27]. Then, the obtained MXene sheets (0.4 g) were dispersed in Fe(NO_3_)_3_·9H_2_O (0.02 M, Fe nitrate, 98.5%, SAMCHUN), polyvinylpyrrolidone (1.0 g, PVP, 40,000, Kanto), polystyrene nanobeads (12 g, PS), in DI water (200 mL), using ultrasonication and stirring. Droplets formed by an ultrasonic humidifier were carried by the N_2_ gas into a tubular reactor to produce Fe_2_O_3_@C/MB (the temperature of the tubular reactor was maintained at 700 °C with a flow rate of 10 L min^−1^). The collected Fe_2_O_3_@C/MB was selenized at 270 °C under an H_2_/Ar atmosphere for 12 h at a ramping rate of 2 °C min^−1^, to form FeSe_*x*_@C/MB. For comparison, the 3D MBs decorated with only iron selenides (FeSe_*x*_/MB) were synthesized by the same process as FeSe_*x*_@C/MB, except for the application of PVP as a carbon source. Bare FeSe_2_–Fe_2_O_3_ and 3D MBs were also prepared by the same procedure from spray solutions with iron salt and MXene nanosheets, respectively. The characterization and electrochemical measurements of the synthesized samples are provided in the Electronic Supplementary Material.

## Results and Discussion

### Synthesis and Characterization of FeSe_***x***_@C/MB, FeSe_***x***_/MB, MB, and Bare FeSe_2_–Fe_2_O_3_

**Scheme 1:**
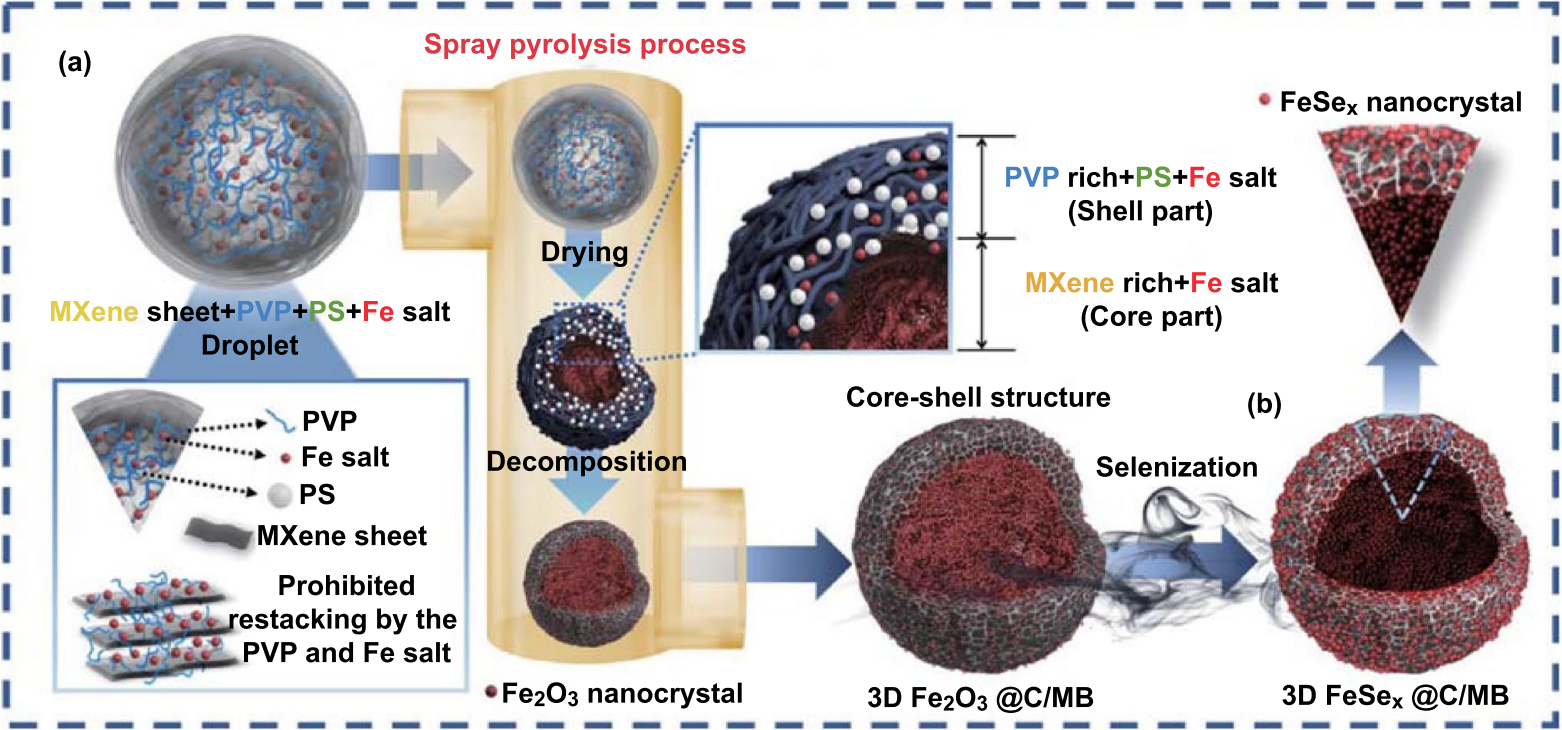
Formation mechanism of FeSe_*x*_@C/MB by a facile two-step strategy

The formation mechanism of FeSe_*x*_@C/MB using a facile two-step strategy is illustrated in Scheme 1. 3D Fe_2_O_3_@C/MB was directly fabricated from a colloidal solution droplet containing MXene nanosheets, polystyrene (PS) nanobeads, polyvinylpyrrolidone (PVP), and Fe nitrate by spray pyrolysis (Scheme 1a). Owing to the small size of the aerosol droplets, rapid evaporation of the solvent occurred upon heating, inducing a strong inward capillary force. This allowed the restructuring of the MXene nanosheets into a 3D structure after complete drying of the solvent. Simultaneously, the phase separation of PVP occurred owing to its low melting point (~ 180 °C), which caused most of the PVP to be present in the outer region of the dried microspheres. As the dried microspheres were continuously heated while passing through the reactor, carbonization of PVP and decomposition of PS nanobeads and Fe nitrate occurred, resulting in 3D Fe_2_O_3_@C/MB. The MXene balls formed from the high-stiffness nanosheets by spray pyrolysis had plentiful polygonal empty voids. After selenization under a reducing atmosphere, 3D Fe_2_O_3_@C/MB transformed into FeSe_x_@C/MB, in which ultrafine iron selenide nanocrystals were uniformly decorated within the C/MB matrix (Scheme 1b). The formation schemes of FeSe_*x*_/MB, FeSe_2_–Fe_2_O_3_, and MB as comparison samples are shown in Fig. S1. Porous Fe_2_O_3_/MB and densely structured Fe_2_O_3_ microspheres formed by spray pyrolysis transformed into FeSe_x_/MB with grown FeSe_*x*_ crystals and FeSe_2_–Fe_2_O_3_ composites by selenization, respectively (Fig. S1a, b). 3D MB microspheres were formed by the one-step spray pyrolysis process from a colloidal solution of MXene nanosheets and PS nanobeads. Restacking of exfoliated 2D MXene nanosheets occurred even within droplets several micrometers in size, as shown in Fig. S1c. However, PVP and Fe-nitrate prevented the restacking of exfoliated 2D MXene nanosheets during the drying stage of the droplets, as described in Scheme 1a.

**Fig. 1:**
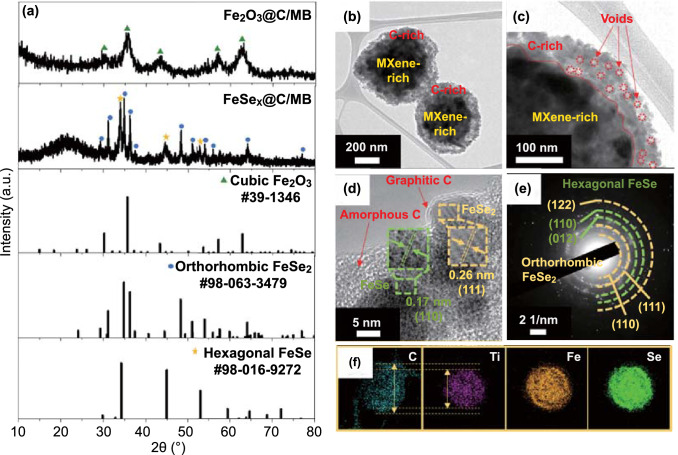
**a** XRD patterns of Fe_2_O_3_@C/MB and FeSe_*x*_@C/MB, **b, c** TEM images, **d** HR-TEM image, **e** SAED pattern, and **f** elemental mapping images of FeSe_*x*_@C/MB

The overall morphologies of 3D Fe_2_O_3_@C/MB and FeSe_*x*_@C/MB are shown in Fig. S2. Regardless of the selenization process, the composite microspheres had a spherical shape with a slightly deflated structure, owing to the shrinkage of the MXene nanosheets during the spray pyrolysis process. Numerous pores were clearly observed on the outer layer of 3D Fe_2_O_3_@C/MB (Fig. S2a, b), which could be owing to the thermal decomposition of the PS nanobeads within the MXene-free layer. In addition, the unique core–shell structure of the composites was confirmed by the distinct contrast between the outer and inner regions, as indicated by the red dashed circle. The X-ray diffraction (XRD) data (Fig. 1a**)** confirmed the transformation of cubic Fe_2_O_3_ nanocrystals into iron selenide nanocrystals of hexagonal FeSe and orthorhombic FeSe_2_ mixed phases. The characteristic peaks of MXene were not observed in the XRD patterns of 3D Fe_2_O_3_@C/MB and FeSe_*x*_@C/MB, owing to the presence of FeSe_*x*_ nanoparticles decorated on MXene. Instead, from the XRD pattern of MB, it is confirmed that the intrinsic crystal phase of MXene hardly changed during the spray pyrolysis process (Fig. S3). Furthermore, in the inset of Fig. S3, the (002) peak related to the stacking of MXene layers is shifted toward smaller angles, implying an expanded layer of MXene during the spray pyrolysis process [28, 29]. Additionally, to verify the presence of MXene in FeSe_*x*_@C/MB, a new sample was prepared by reducing the amounts of the Fe nitrate and PVP to 1/10 while maintaining that of MXene (denoted as FeSe_*x*_@C/MB-1/10), and then was analyzed by the XRD. As can be seen in Fig. S4a, the surface roughness of FeSe_*x*_@C/MB-1/10 was slightly different from that of the original FeSe_*x*_@C/MB, which was closer to that of 3D MB due to the reduced amounts of Fe nitrate and PVP. In the XRD pattern (Fig. S4b) a distinct peak corresponding to the (002) plane was observed at 8.3°, which is related to the stacking of MXene layers. The board peak confirmed at 20–25° could be attributed to SiO_2_ holder for XRD the measurement. In light of these results, it is considered that the diffraction peaks of MXene were not observed in the XRD pattern of FeSe_*x*_@C/MB due to the relatively large amount of FeSe_2_ and its high crystallinity.

The transmission electron microscopy (TEM) image of FeSe_*x*_@C/MB showed a core–shell structure with numerous voids on the outer layer (Fig. 1b). From the magnified TEM image (Fig. 1c), a distinct contrast between the inner and outer regions was confirmed. The measured shell thickness was approximately 70 nm. High-resolution TEM (HR-TEM) images revealed that the FeSe_*x*_ nanoparticles were well embedded in the amorphous carbon matrix formed by the carbonization of PVP upon the spray pyrolysis process (Fig. 1d). Interestingly, despite the relatively low synthesis temperature, graphitic layers were identified on the surfaces of some particles, which could be owing to the catalytic effect of metallic Fe nanocrystals formed during the selenization process. In addition, the lattice distances of 0.26 and 0.17 nm were confirmed for the crystalline particles, corresponding to the (111) and (110) planes of FeSe_2_ and FeSe crystal phases, respectively. The results of selected area electron diffraction (SAED) data in Fig. 1e reveal that the nanoparticles had a dominant orthorhombic FeSe_2_ crystal phase and a minor hexagonal FeSe crystal phase without other impurities. Notably, no patterns related to MXene were identified in the SAED results. Instead, the existence of MXene was clearly confirmed by the elemental mapping images (Fig. 1f). These results show that C, Fe, and Se elements were uniformly distributed over the microspheres, while most Ti elements were only detected in the inner region, indicating that most of the MXene was present inside the microspheres. Furthermore, for accurate comparison, the mapping images of C and Ti element were superimposed as follow (Fig. S5).

**Fig. 2:**
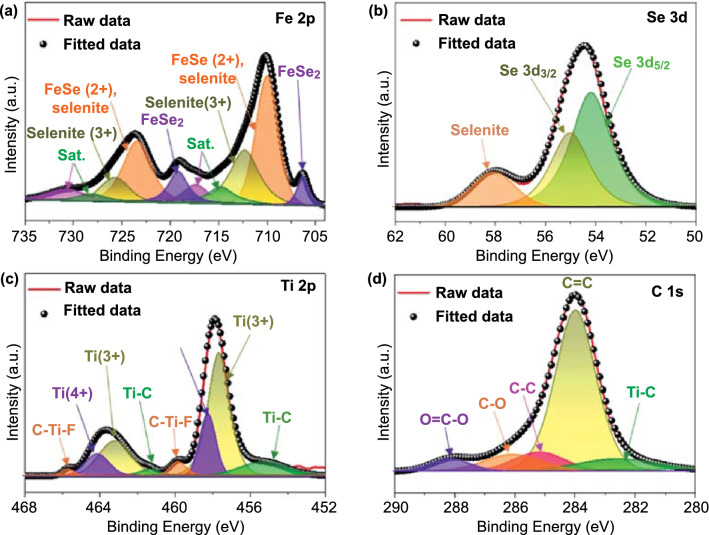
XPS spectra of FeSe_*x*_@C/MB: **a** Fe 2p, **b** Se 3d, **c** Ti 2p, and **d** C 1s spectra

X-ray photoelectron spectroscopy (XPS) analysis also revealed the coexistence of C, Ti, Fe, Se, and O elements in 3D FeSe_*x*_@C/MB (Fig. S6). The high-resolution Fe 2p spectrum (Fig. 2a) was resolved into three pairs of doublets for FeSe_2_ (706.3/719.2 eV), FeSe/selenite (2 +) (710.0/723.4 eV), and selenite (3 +) (712.3/725.7 eV), along with weak peaks from the satellites (714.8 and 717.3 eV/728.2 and 730.7 eV) [30, 31]. The Se 3d spectrum (Fig. 2b) showed three different peaks of Se 3d_5/2_, Se 3d_3/2_, and selenite. The first two peaks were attributed to iron selenides, and the other one was owing to the partial oxidation of 3D FeSe_*x*_@C/MB [32, 33]. The Ti 2p spectrum (Fig. 2c) showed multiple component peaks corresponding to Ti-C, Ti^3+^_,_ Ti^4+^, and C–Ti–F bonds, indicating that the MXene nanosheets with the chemical formula of Ti_n+1_C_n_F_x_ were partially oxidized during the spray pyrolysis process [34–37]. The C 1s (Fig. 2d) spectrum could be deconvoluted into five peaks for Ti-C (282.5 eV), C=C (284.0 eV), C–C (285.2 eV), C-O (286.1 eV), and O=C–O (288.1 eV) bonds. The presence of the Ti-C bond was ascribed to MXene [37, 38].

**Fig. 3:**
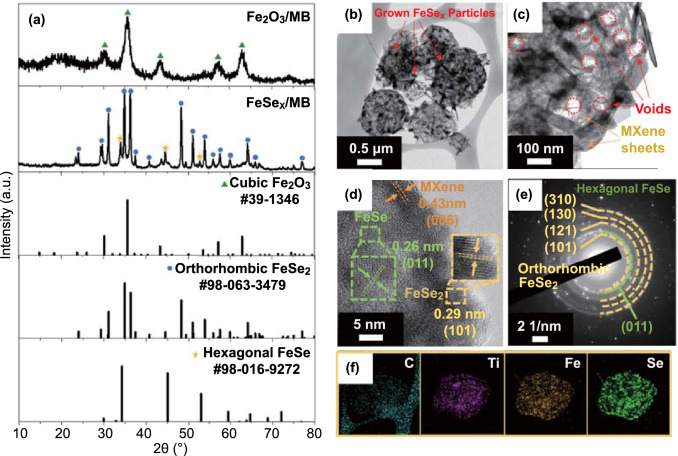
**a** XRD patterns of Fe_2_O_3_/MB and FeSe_*x*_/MB, **b, c** TEM images, **d** HR-TEM image, **e** SAED pattern, and **f** elemental mapping images of FeSe_*x*_/MB

In the case of 3D Fe_2_O_3_/MB prepared without adding PVP, no outer pores or core–shell structures were identified, indicating that PVP plays an important role in controlling the structure of the composites (Fig. S7a, b). After selenization under a reducing atmosphere, the Fe_2_O_3_ nanoparticles were transformed into FeSe_*x*_ nanoparticles, forming 3D FeSe_*x*_/MB. The morphology of 3D FeSe_*x*_/MB was similar to that of 3D FeSe_*x*_@C/MB. However, unlike 3D FeSe_*x*_@C/MB, 3D FeSe_*x*_/MB consisted of rod-shaped nanoparticles that were the result of the overgrowth of FeSe_*x*_ owing to the Ostwald ripening (Fig. S7c, d) [39]. Although 3D Fe_2_O_3_@C/MB and Fe_2_O_3_/MB had different structural features, the XRD patterns of Fe_2_O_3_/MB corresponded to the crystal phases of cubic Fe_2_O_3_, and Fe_2_O_3_ nanocrystals transformed into iron selenide nanocrystals of hexagonal FeSe and orthorhombic FeSe_2_ mixed phases after selenization (Fig. 3a).

As shown in Fig. 3b, the FeSe_*x*_ nanorods had irregular sizes and morphologies, and mostly exposed on the surface of the 3D MXene balls. In light of these results, the carbon matrix in 3D FeSe_*x*_@C/MB effectively inhibited the overgrowth of FeSe_*x*_ during the selenization process. TEM image (Fig. 3c) showed clear empty voids owing to the stiffness of the MXene nanosheets and PS nanobeads. HR-TEM imaging (Fig. 3d) confirmed the lattice fringes with distances of 0.29, 0.26, and 0.43 nm, which were indexed to the (101), (011), and (006) planes of FeSe_2_, FeSe, and MXene, respectively. The XRD and SAED patterns of 3D FeSe_*x*_/MB were similar to those of 3D FeSe_*x*_@C/MB (Fig. 3a, e). The mapping images of C, Ti, Fe, and Se overlapped well with each other (Fig. 3f).

A 3D MXene ball with numerous surface ridges was also synthesized as a comparison sample by spray pyrolysis of a spray solution containing only MXene nanosheets (Fig. S8). The MXene ball reported previously exhibited a typical 2D layer with a lateral size of several tens of micrometers. On the other hand, similar structured reduced graphene oxide balls did not show a clear 2D layer. The stiffness of the 2D MXene nanosheets (Fig. S8a, b) resulted in a uniquely structured 3D ball with plentiful polygonal empty voids (Fig. S8c, d). HR-TEM imaging confirmed a few layers separated by 1.26 and 0.15 nm, corresponding to the (002) and (110) planes of the MXene nanosheets (Fig. S8e). Elemental mapping images (Fig. S8f) exhibited an even distribution of C, Ti, and O throughout the 3D MXene ball.

The synthesis of bare FeSe_*x*_ microspheres as a comparison sample was also attempted by selenization of Fe_2_O_3_ microspheres, which were synthesized by spray pyrolysis from a spray solution with only Fe nitrate. The scanning electron microscopy (SEM) images (Fig. S9a, b) show that the surfaces of the polydisperse microspheres became rougher than before selenization. However, FeSe_2_–Fe_2_O_3_ composite microspheres were formed by the incomplete selenization of dense Fe_2_O_3_ microspheres. As can be seen in the XRD patterns (Fig. S9c), the microspheres exhibited a typical Fe_2_O_3_ crystalline phase before the selenization, but after the selenization, two different crystal phases (orthorhombic FeSe_2_ and hexagonal Fe_2_O_3_) were observed. The incomplete phase transformation into the metal selenide could be owing to the dense structure of the FeSe_2_–Fe_2_O_3_ microspheres, which precluded the H_2_Se gas from penetrating into the microspheres.

The N_2_ adsorption–desorption isotherm results revealed that the 3D MXene ball had a higher specific surface area (64.8 m^2^ g^−1^) relative to pristine 2D MXene (7.6 m^2^ g^−1^), proving that 3D structuring can effectively inhibit the restacking of MXene (Fig. S10a). In addition, compared with the 3D MXene ball, the slightly reduced specific surface areas of 3D FeSe_*x*_/MB (52.9 m^2^ g^−1^) and FeSe_*x*_@C/MB (22.7 m^2^ g^−1^) could be ascribed to the presence of FeSe_*x*_ crystals and carbon matrix (Fig. S10b). Meanwhile, the FeSe_2_–Fe_2_O_3_ microspheres exhibited an extremely small specific surface area (0.2 m^2^ g^−1^), owing to their dense structure. From the Barrett–Joyner–Halenda (BJH) pore-size distribution data (Fig. S10c, d), 3D MXene-based materials were confirmed to have well-developed meso- and macroporous structures, consistent with the SEM and TEM results.

**Fig. 4:**
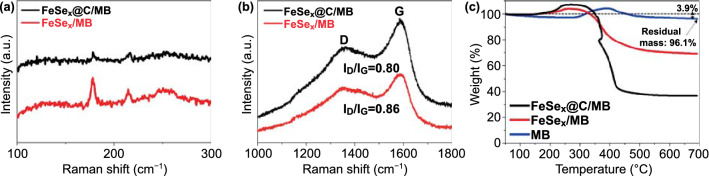
**a, b** Raman spectra of FeSe_*x*_@C/MB and FeSe_*x*_/MB and **c** TG curves of FeSe_*x*_@C/MB, FeSe_*x*_/MB, and MB

Figure 4 displays the Raman spectra of the 3D FeSe_*x*_/MB and FeSe_*x*_@C/MB composites and TG curves of FeSe_*x*_@C/MB, FeSe_*x*_/MB, and MB. In Fig. 4a, the 3D FeSe_*x*_/MB and FeSe_*x*_@C/MB samples exhibited three peaks (178.1, 214.4, and 250.2 cm^−1^) in the 100–300 cm^−1^ region. Based on previous reports, the peak at 214.4 cm^−1^ was a Se-Se stretching mode, and the peaks at 178.1 and 250.2 cm^−1^ were Se-Se librational and stretching vibrations or their combination of Fe-Se bonds, respectively [40, 41]. In addition, the D and G bands in the 1200–1800 cm^−1^ region indicate the presence of amorphous carbon with numerous defects in the composites (Fig. 4b) [42, 43]. Interestingly, even though no carbon source was added, the D and G bands were identified in the FeSe_*x*_/MB sample, which could be owing to the reordering of C atoms lost in MXene during the annealing, as reported in previous studies [44].

In the thermal gravimetric analysis (TGA) results (Fig. 4c), the 3D MB sample showed an initial weight increase for temperatures in the 300–400 °C range, followed by a weight loss, which could be attributed to the oxidation of MXene into TiO_2_ and to the release of the CO_2_ gas [45]. The final weight loss of the 3D MB sample was 3.9%. Meanwhile, the TGA curves of FeSe_*x*_/MB and FeSe_*x*_@C/MB showed a similar initial weight increase for temperatures in the 250–300 °C range, corresponding to a partial oxidation of metal selenides to metal selenates and SeO_2_ [46]. The subsequent weight loss could be attributed to the combustion of carbonaceous material and further oxidation of metal selenides to metal oxides. Due to the presence of PVP derived carbon, FeSe_*x*_@C/MB exhibited greater weight loss for temperatures in the 300–400 °C. Based on these results, the carbon content of the 3D FeSe_*x*_@C/MB composite was determined to be 32.5%.

### Investigation of K-Ion Storage Mechanism and KIB Performances

**Fig. 5:**
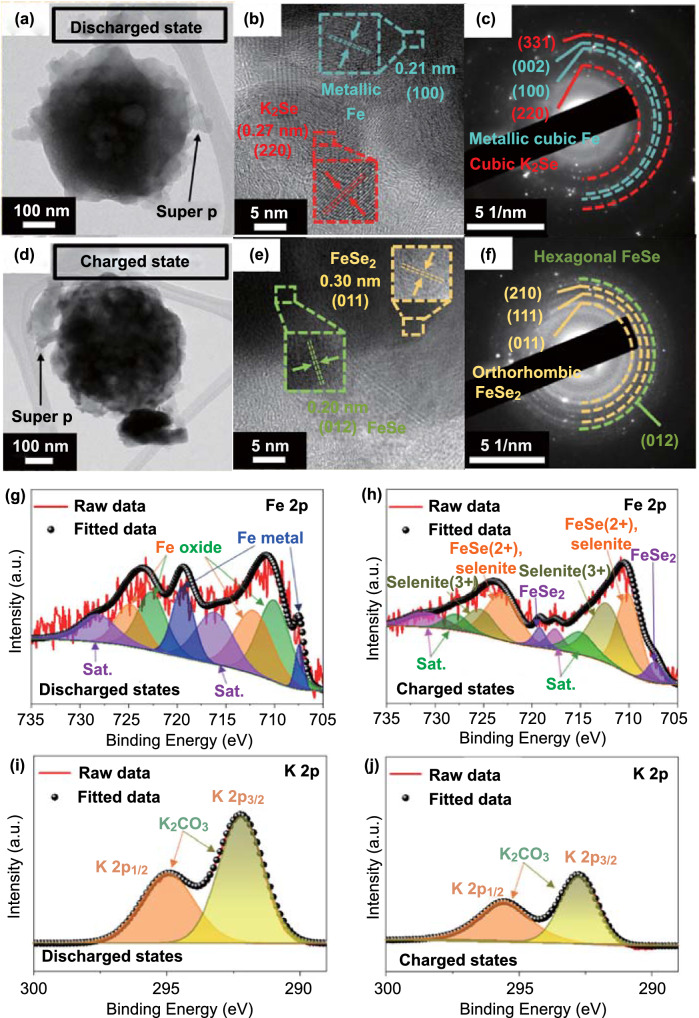
*Ex-situ* TEM images, SAED patterns, XPS spectra of FeSe_*x*_@C/MB after the first discharge (**a–c, g, i)** and charge state (**d–f, h, j**): **a, d** TEM images, **b, e** HR-TEM images, **c, f** SAED patterns, **g, h** Fe 2p, and **i, j** K 2p spectra

To confirm the electrochemical conversion mechanism of FeSe_*x*_@C/MB during potassiation/depotassiation, *ex-situ* TEM and XPS analyses were conducted at the first discharged and charged states (Fig. 5). In the *ex-situ* TEM images (Fig. 5a, d), the overall morphology of the composite was well maintained, but the detailed structure could not be verified owing to the presence of a polymeric layer formed after the cycling. After the fully discharged state, HR-TEM imaging (Fig. 5b) revealed the crystal lattice fringes separated by 0.21 and 0.27 nm, which were indexed to the (100) plane of metal cubic Fe and to the (220) plane of cubic K_2_Se, respectively. The relevant rings in the SAED pattern (Fig. 5c) also matched well the metal cubic Fe and K_2_Se. These results suggest that iron selenides in FeSe_*x*_@C/MB are converted into metal Fe nanoparticles after full discharge. At that time, the K_2_Se by-product was also formed through the reaction between potassium and Se ions. In the *ex-situ* XRD results of the MB electrode measured after the initial discharge, the (002) peak related to the stacking of MXene layers was barely verified, indicating the expansion of the MXene layer owing to the intercalation of K^+^ (Fig. S11) [47]. Meanwhile, the HR-TEM image of FeSe_*x*_@C/MB in the fully charged state showed the existence of orthorhombic FeSe_2_ and hexagonal FeSe (Fig. 5e). Furthermore, the (002) peak was still not confirmed in the *ex-situ* XRD data after the initial charge. This result suggests that the expanded layers of MXene remained after the extraction of K^+^ ions [47], and metal Fe nanoparticles returned to the corresponding iron selenides after the charge process. The corresponding SAED pattern rings (Fig. 5f) also show the formation of FeSe_*x*_ crystals in a reversible conversion reaction.

In the XPS results, the high-resolution Fe 2p spectrum of FeSe_*x*_@C/MB in the fully discharged state shows paired deconvoluted peaks corresponding to Fe metal nanoparticles as well as iron oxide (Fig. 5g). The presence of the oxidized state can be attributed to the properties of metallic particles that are prone to oxidation. Notably, these peaks disappeared after full charging (Fig. 5h), and new peaks appeared with weakened crystallinity. In the K 2p spectrum, the intensity of the peak corresponding to K_2_CO_3_ was higher in the fully discharged state than in the fully charged state, which could be ascribed to the solid electrolyte interphase (SEI) layer, composed of K_2_CO_3_, that became partially decomposed after the charging process (Fig. 5i, j) [48, 49]. These electrochemical conversions also confirm the above-mentioned *ex-situ* TEM results.

**Fig. 6:**
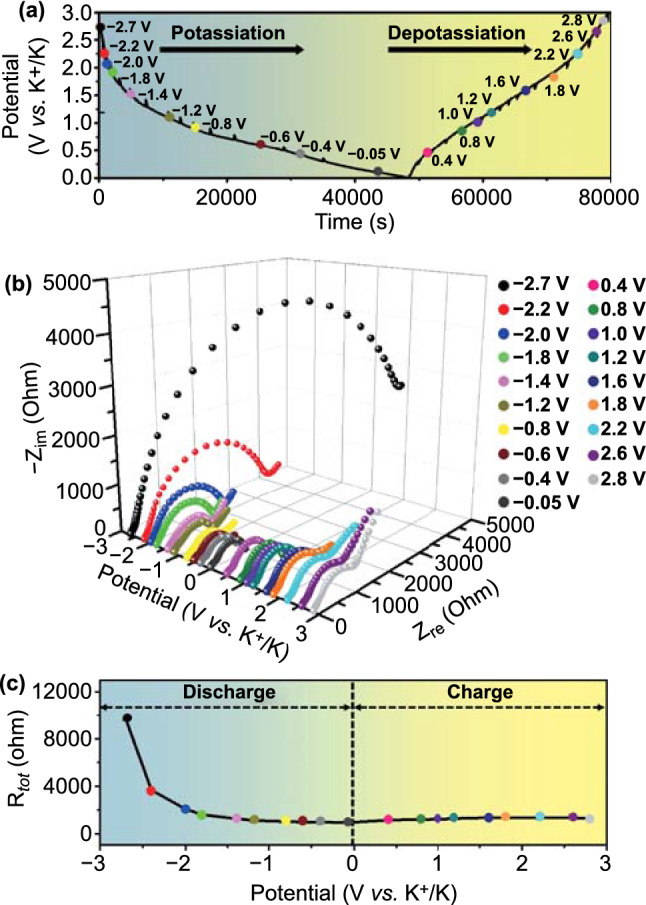
*In-situ* EIS of FeSe_*x*_@C/MB: **a** Potential *vs.* times curve, **b** *in-situ* Nyquist plots, and **c** R_tot_ values obtained at pre-selected potentials during the first cycles at 0.05 A g^−1^

The electrochemical impendence spectroscopy (EIS) analysis was carried out *in-situ* during the initial cycle, at a scan rate of 0.05 A g^−1^, to support the electrochemical conversion mechanism of FeSe_*x*_@C/MB. In Fig. 6a, the data points indicate the preselected potentials for *in-situ* EIS measurements during potassiation/depotassiation at 0.05 A g^−1^. The Nyquist plots fitted by the Randle-type equivalent circuit (Fig. S12) and the change in R_tot_ (sum of interfacial resistances related to the SEI (R_sei_) and to the charge transfer resistance (R_ct_)) are shown in Fig. 6b and c. During the first discharge process, R_tot_ continuously decreased from the potential to -0.05 V, owing to ultrafine metal Fe nanoparticles with high electrical conductivity, as confirmed by the *ex-situ* TEM analysis (Fig. 5). During the subsequent charging process, R_tot_ gradually increased until 1.8 V, owing to the structural stress and the phase transformation into FeSe_*x*_, which has a lower electrical conductivity than metallic Fe. Beyond 1.8 V, the reduction of R_tot_ could be owing to the volume contraction induced complete depotassiation and partial decomposition of the SEI layer [50], as confirmed by the *ex-situ* XPS (Fig. 5i, j).

**Fig. 7:**
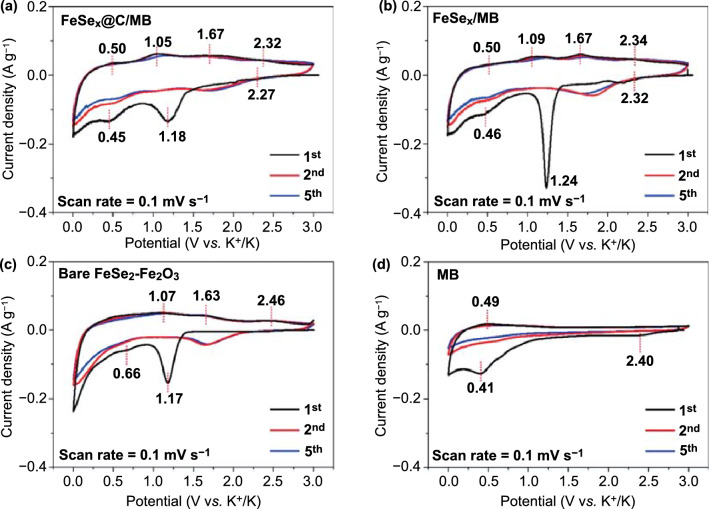
CV data at voltage range 0.001–3.0 V: **a** FeSe_*x*_@C/MB, **b** FeSe_*x*_/MB, **c** bare FeSe_2_–Fe_2_O_3_, and **d** MB

Cyclic voltammetry (CV) was conducted at a scan rate of 0.1 mV s^−1^, for potentials in the 0.001–3.0 V range (*vs.* K^+^/K) for the first, second, and fifth cycles, to investigate the electrochemical conversion characteristics of the FeSe_*x*_@C/MB, FeSe_*x*_/MB, FeSe_2_–Fe_2_O_3_, and MB electrodes (Fig. 7a–d). In the first cathodic sweep, FeSe_*x*_@C/MB exhibited broad three peaks, in which the peak observed at 2.27 V was associated with the intercalation of K^+^ into MB [15], and peaks at 1.18 and 0.45 V corresponded to the intercalation of K^+^ into FeSe_*x*_ and the formation of metal Fe and K_2_Se from K_*x*_FeSe_*y*_, respectively [51, 52]. In addition, during the subsequent cathodic sweeps, the initial peaks disappeared, and new peaks appeared at a higher potential with low intensity. These peaks imply the irreversible formation of an SEI layer during the first discharge process [53]. In the case of the first anodic sweep, broad peaks were observed at 0.50, 1.05, 1.67, and 2.32 V for FeSe_x_@C/MB. The peak at 0.50 V was related to the extraction of K^+^ from MB [15], and the peaks at 1.05, 1.67, and 2.32 V were attributed to the transformation of metallic Fe nanoparticles and K_2_Se into FeSe_*x*_ nanocrystals [54, 55]. The CV data of FeSe_*x*_/MB showed similar tendencies to those of FeSe_*x*_C/MB, but the peaks were more apparent than in the case of FeSe_*x*_@C/MB, owing to the formation of larger FeSe_*x*_ crystals in the absence of carbon. On the other hand, CV-shaped FeSe_2_–Fe_2_O_3_ microspheres exhibited slightly different trends from those of FeSe_*x*_@C/MB and FeSe_*x*_@C, featuring a peak at 0.66 V, which corresponded to the conversion reaction of Fe_2_O_3_ [56, 57]. In addition, the area of the CV curve for the FeSe_2_–Fe_2_O_3_ microspheres was smaller than those for FeSe_*x*_@C/MB and FeSe_*x*_/MB, implying a lower specific capacity of the FeSe_2_–Fe_2_O_3_ microspheres. Meanwhile, MBs exhibited two cathodic peaks at 2.40 and 0.41 V for the first cathodic sweep, which corresponded to the K^+^ intercalation reaction and to the formation of the SEI layer, respectively [15, 58]. According to a previous study [17, 59], a high potential is related to an irreversible reaction between the surface functional groups of MXene and the electrolyte, while low-potential peaks are related to the K^+^ intercalation in the small interlayer space with the formation of the SEI layer. Moreover, the cathodic peak located in the range of low potentials persisted even after the first sweep, indicating the reversible intercalation and stability of the MXene phase after repeated potassiation/depotassisation.

**Fig. 8:**
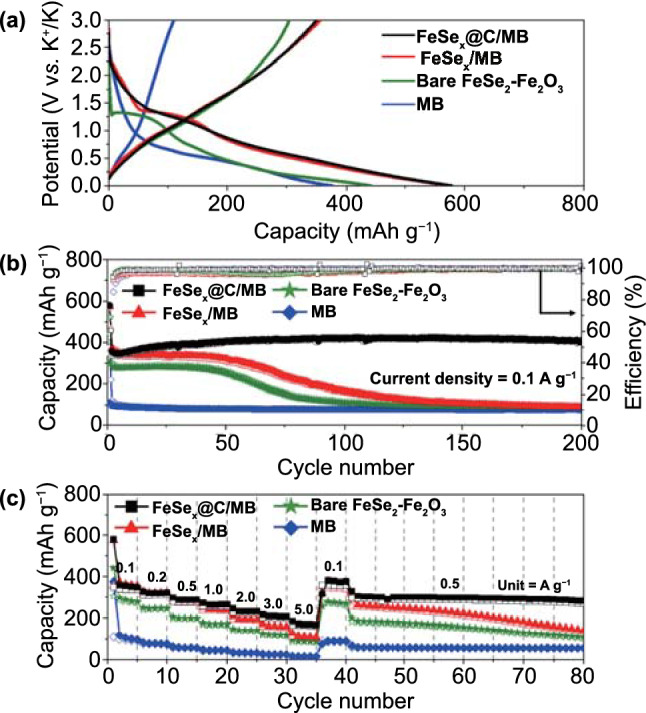
Electrochemical properties of FeSe_*x*_@C/MB, FeSe_*x*_/MB, bare FeSe_2_–Fe_2_O_3_, and MB: **a** initial charge–discharge curves, **b** cycle performances at a current density 0.1 A g^−1^, and **c** rate performances at various current densities

To probe the K-ion storage performance of FeSe_*x*_@C/MB, FeSe_*x*_/MB, FeSe_2_–Fe_2_O_3_, and MB electrodes, a galvanostatic charge–discharge test was conducted (Fig. 8). The initial charge–discharge profiles for the four electrodes were obtained at a current density of 0.1 A g^−1^, and are shown in Fig. 8a. Distinct plateaus were confirmed in the 1.18–1.30 V range for the initial discharge curves of FeSe_*x*_@C/MB, FeSe_x_/MB, and FeSe_2_–Fe_2_O_3_ electrodes. In the case of MB, a plateau corresponding to the intercalation reaction between MXene and K^+^ was observed for lower potentials (0.40–0.50 V), in agreement with the CV results. The initial discharge/charge capacities of the FeSe_*x*_@C/MB, FeSe_x_/MB, FeSe_2_–Fe_2_O_3_, and MB electrodes were 579/350, 574/356, 441/304, and 376/109 mAh g^−1^, and their Coulombic efficiencies (CEs) were 60%, 62%, 69%, and 29%, respectively. Compared with FeSe_*x*_/MB, the lower initial CE of FeSe_*x*_@C/MB could be attributed to the presence of disordered carbon material with a high initial irreversible capacity [60, 61]. Meanwhile, the lowest initial CE of MB was attributed to its high specific surface area, which provided more sites for the formation of an irreversible SEI layer.

Figure 8b shows the cycling performance of the four electrodes, assessed at a current density of 0.1 A g^−1^. The discharge capacities of the FeSe_*x*_@C/MB, FeSe_*x*_/MB, FeSe_2_–Fe_2_O_3_, and MB electrodes after 200 cycles were 410, 89, 89, and 76 mAh g^−1^, respectively. The capacity of the FeSe_*x*_@C/MB electrode gradually increased during the first 50 cycles owing to the electrode activation, but then settled at a certain level and remained so without significant degradation until 200 cycles. Moreover, the CEs of FeSe_x_@C/MB quickly reached 99% within 10 cycles, and remained at that level during the entire cycle process. On the other hand, FeSe_*x*_/MB exhibited a rapid capacity decay after 50 cycles and eventually attained capacity similar to that of FeSe_2_–Fe_2_O_3_. Nevertheless, the SEM images of the two electrodes after 200 cycles revealed that the original spherical morphologies of the FeSe_*x*_@C/MB and FeSe_*x*_/MB composites were well retained, without severe structural collapse (Fig. S13a, b). These results indicate that the exposed FeSe_*x*_ nanoparticles without carbon coating were easily pulverized during cycling, while the overall morphology of the composites did not collapse owing to the presence of MXene. In previous studies [27, 62], it was noted that this pulverization can occur owing to the different volume expansion rates of active materials and MXene and the weak interaction between them. Meanwhile, the bare FeSe_2_–Fe_2_O_3_ microspheres exhibited a relatively low reversible capacity during the early cycles, owing to the presence of Fe_2_O_3_ crystals with poor K-ion storage performance, and their capacity decreased faster than that of FeSe_*x*_/MB after the 50th cycle. The SEM image of the bare FeSe_2_–Fe_2_O_3_ electrode after 200 cycles shows the pulverized microspheres, indicating that MB could contribute to the structural stability (Fig. S13c). MB exhibited a low specific capacity but excellent cycling stability, for the entire number of cycles.

The rate performances of the four electrodes were evaluated for various current ranges, from 0.1 to 5.0 A g^−1^ (Fig. 8c). FeSe_*x*_@C/MB exhibited a stable reversible capacity over the entire range of current densities and yielded discharge capacities of 349, 322, 287, 263, 230, 206, and 169 mAg h^−1^ at the current densities of 0.1, 0.2, 0.5, 1.0, 2.0, 3.0, and 5.0 A g^−1^, respectively. In addition, when the current density decreased to 0.1 from 5.0 A g^−1^, the capacity of the FeSe_x_@C/MB electrode mostly recovered, indicating that the structural degradation was minimal at high current densities, owing to the electrode’s excellent structural robustness. Furthermore, although the current density increased again to 0.5 A g^−1^, the electrode’s capacity remained high and stable, without any rapid capacity decay. By contrast, FeSe_*x*_/MB exhibited reversible discharge capacities of 357, 323, 277, 237, 191, 158, and 109 mAg h^−1^ at the current densities of 0.1, 0.2, 0.5, 1.0, 2.0, 3.0, and 5.0 A g^−1^, respectively. Although the FeSe_*x*_/MB electrode exhibited reversible capacities similar to those of the FeSe_x_@C/MB electrode at low current densities, its capacity decreased more significantly with increasing current density (compared with the FeSe_x_@C/MB electrode). Moreover, the capacity continued to decrease, even at low current densities. In light of these results, we concluded that the carbon layer effectively inhibits the volume expansion of FeSe_x_ nanoparticles and facilitates electron transport. Compared with previously reported Fe-based anodes for KIBs, the FeSe_x_@C/MB electrode demonstrated superior electrochemical performance, especially in terms of cycling stability and specific capacity (Table S1). The FeSe_2_–Fe_2_O_3_ and MB electrodes exhibited lower specific capacities than the FeSe_*x*_@C/MB and FeSe_*x*_/MB electrodes, at all current densities. Also, to further confirm the advantages of 3D structured MXene for K^+^ storage performance, bare 2D MXene was galvanostatically tested as anodes for KIBs under the same condition as the other electrode. As shown in Fig. S14a, b, the 2D MXene electrode exhibited lower reversible capacity over the entire cycle compared the MB electrode. In particular, the initial discharge capacity (163 mAh g^−1^) of 2D MXene electrode was less than half that of the MB electrode (376 mAh g^−1^). Furthermore, the difference in capacity between the two electrodes remained similar at various current densities from 0.1 to 3.0 A g^−1^ (Fig. S14c). These results indicate that the electrochemical performance of 3D MB electrode is better than that of the 2D MXene electrode, which can be attributed to a larger surface area and enhanced ion transport of 3D MB.

**Fig. 9:**
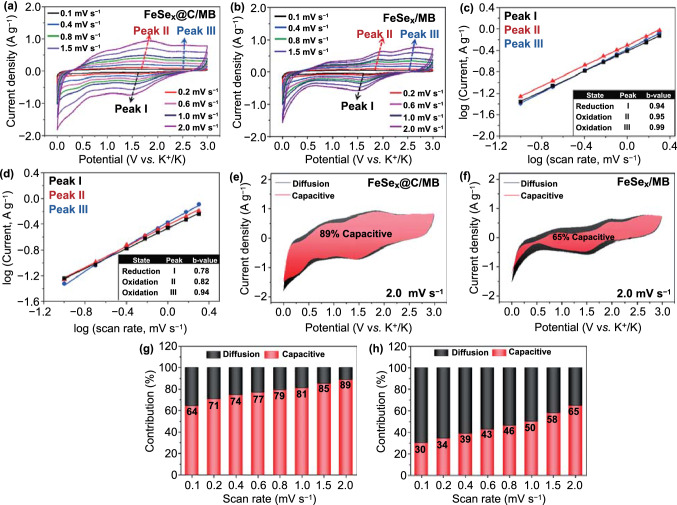
**a, b** Cyclic voltammograms at various sweep rates, **c, d** fitted log (peak current) *vs.* log (scan rate) for peaks corresponding to certain electrochemical reactions, **e, f** cyclic voltammograms showing capacitive contribution (colored area) at a scan rate of 2.0 mV s^−1^, and **g, h** capacity contribution at various scan rates of FeSe_x_@C/MB and FeSe_x_/MB

To elucidate the superior rate performance of FeSe_*x*_@C/MB, CV measurements were carried out at scan rates in the 0.1–2.0 mV s^−1^ range (Fig. 9). As shown in Fig. 9a, b, the redox peaks in the CV curves of FeSe_*x*_@C/MB and FeSe_*x*_/MB gradually shifted with increasing the scan rate, owing to the electrode polarization [53]. The current density values of the apparent redox peaks in the CV curves of the two electrodes (denoted as peaks I and II, respectively) were fitted to logarithmic functions of the scan rate, according to the relationship *i* = *a*ν^*b*^ (where *i* is the measured current, *v* is the scan rate, and both *a* and *b* are experimentally determined parameters) [63]. For *b* close to 1.0, the electrochemical reaction is dominantly controlled by the capacitive behavior [64]. The *b*-values of 0.94, 0.95, and 0.99 respectively, for peaks I, II, and III of FeSe_*x*_@C/MB were higher than those of FeSe_*x*_/MB, as shown in Fig. 9c, d, implying that FeSe_*x*_@C/MB has a more dominant capacitive property. Furthermore, the capacitive contribution values of FeSe_*x*_@C/MB and FeSe_*x*_/MB, calculated based on the previously reported equation [65], gradually increased as the scan rate increased, reaching 89% and 65%, respectively, for the scan rate of 2.0 mV s^−1^ (Fig. 9e–h). These high capacitive contributions represent the rapid electron transfer of FeSe_x_@C/MB and support the rationale for its outstanding rate performance.

**Fig. 10:**
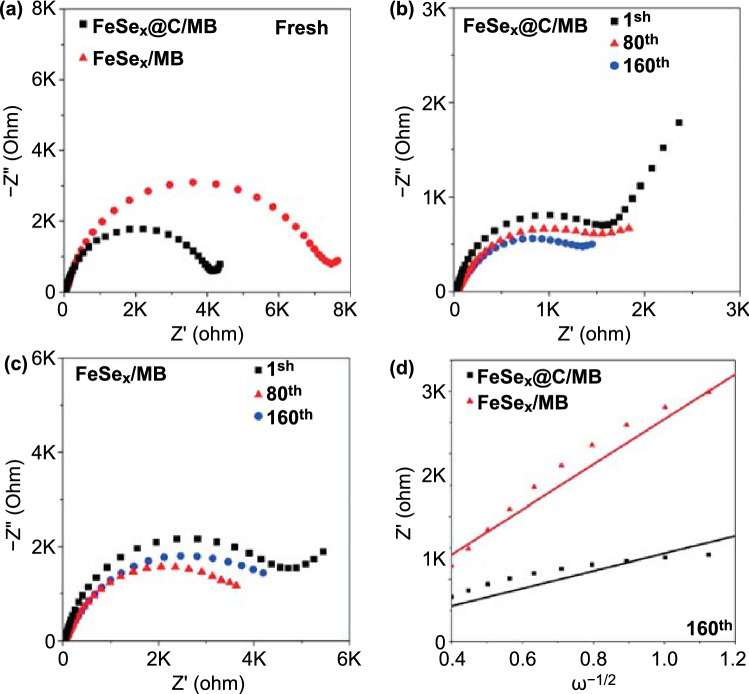
Nyquist plots of of FeSe_*x*_@C/MB and FeSe_x_/MB: **a** fresh cells, **b, c** after the 1st, 80th, and 160th cycle, and **d** the relationship between the phase angle (ω^−1/2^) and impedance (Z’) at the 160th cycle

*Ex-situ* EIS measurements were performed to understand the charge transfer kinetics of the electrodes (Fig. 10). In the fresh state (Fig. 10a), the FeSe_*x*_@C/MB electrode exhibited lower R_ct_ than the FeSe_x_/MB electrode, indicating better charge transfer kinetics of the FeSe_*x*_@C/MB electrode. After the first cycle, the R_ct_ values of the both electrodes decreased, which could be owing to the formation of ultrafine FeSe_x_ nanocrystals and electrode activation (Fig. 10b, c). Notably, the R_ct_ values of the FeSe_x_@C/MB electrode changed slightly as the cycling progressed, while those of FeSe_x_/MB increased significantly after 160 cycles. These results arise from the increase in resistance owing to the pulverization of FeSe_x_ nanoparticles without the carbon coating in the FeSe_*x*_/MB electrode during cycling. These results were in a good agreement with the cycle data (Fig. 8b). The linear slope in the low-frequency region of the Nyquist plot is associated with the Warburg impedance of potassium-ions’ diffusion [66]. The relationship between the square root of the angular frequency (ω^−1/2^) and the real part impedance (Z’) of the two electrodes cycled at the 160th cycle is presented in Fig. 10d. Compared with the result for the FeSe_*x*_/MB electrode, the lower slope for the FeSe_x_@C/MB electrode implies a faster diffusion of K^+^ ions after 160 cycles.

## Conclusions

In summary, we report a novel strategy for designing carbon-coated 3D MXene/iron selenide balls with aggregation-resistant properties, for achieving high-performance KIBs. Using the ultrasonic spray pyrolysis method, 2D MXene nanosheets were easily converted into a corresponding 3D ball by rapid evaporation of droplets. The 3D MXene architectures featured a larger surface area, higher mechanical strength, and better accessibility for electrolytes and ions, while maintaining the attractive properties of MXene. Furthermore, combining iron selenides and carbon with 3D MXene balls offered many more sites for ion storage and enhanced the structural robustness of composite balls. Accordingly, the FeSe_x_@C/MB anode exhibited a high reversible capacity, long cycling stability, and an excellent rate capability, when used in KIBs. We believe that our strategy of synthesizing 3D MXene-based composites can also be using for preparing other electrode materials for utilization in high-performance energy storage applications.

## Supplementary Information

Below is the link to the electronic supplementary material.

Supplementary file1 (DOCX 8545 kb)
