# Supplementary material for: Carbon-Coated Three-Dimensional MXene/Iron Selenide Ball with Core–Shell Structure for High-Performance Potassium-Ion Batteries
Source: Nanomicro Lett. 2021 Dec 6;14:17. doi: 10.1007/s40820-021-00741-0 (PMC8648910; doi:10.1007/s40820-021-00741-0)
Supplement: Supplementary file 1 — Supplementary file1 (DOCX 8545 kb) [file 40820_2021_741_MOESM1_ESM.docx]

Supporting Information for

**Carbon-Coated Three-Dimensional MXene/Iron Selenide Ball with Core-Shell Structure for High-Performance Potassium-Ion Batteries**

Su Hyun Yang^1^, Yun Jae Lee^2^, Heemin Kang^1^, Seung-Keun Park^2,^*, and Yun Chan Kang^1,^*

^1^Department of Materials Science and Engineering, Korea University, Anam-Dong, Seongbuk-Gu, Seoul 136-713, Republic of Korea

^2^Department of Advanced Materials Engineering, Chung-Ang University, 4726 Seodong-daero, Daedeok-myeon, Anseong-si, Gyeonggi-do, 17546, Republic of Korea

*Corresponding authors E-mail: yckang@korea.ac.kr (Prof. Y.C. Kang) and skpark09@cau.ac.kr (Prof. S.-K. Park)

**Supplementary Characterizations of Materials**

The morphologies of the prepared FeSe_x_@C/MB, FeSe_x_/MB, FeSe_2_-Fe_2_O_3_ microspheres, and MBs were examined using microscopic characterization techniques, including scanning electron microscopy (SEM, VEGA3 SBH) and field-emission transmission electron microscopy (FE-TEM, JEM-2100 F). X-ray photoelectron spectroscopy (XPS, Thermo Scientific K-Alpha) was conducted to confirm the chemical nature of FeSe_x_@C/MB. The crystal phases of the prepared samples were characterized by powder X-ray diffraction (XRD, X’Pert PRO) with Cu-Kα radiation (λ = 1.5418 Å), at the Korea Basic Science Institute (Daegu Center). Their surface areas and pore sizes were investigated using the Brunauer–Emmett–Teller (BET) method, with pure N_2_ as the adsorbate gas. Thermogravimetric analysis (TGA) was performed using a Pyris 1 TGA (Perkin Elmer) in the 30–700 °C range, at a ramp rate of 10 °C·min^−1^ in air, to confirm the carbon content of the composites. Raman spectroscopy (Jobin Yvon LabRamHR800, excited by a 632.8-nm-wavelength He/Ne laser) was performed, for analyzing the structure of the carbon and Se bonding of FeSe_x_ in FeSe_x_@C/MB and FeSe_x_/MB.

**Supplementary Electrochemical Measurements**

The electrochemical properties of FeSe_x_@C/MB, FeSe_x_/MB, FeSe_2_-Fe_2_O_3_ microspheres, and MBs were evaluated using a standard 2032-type coin cell. The anodes were prepared by mixing active material (70 wt%), Super P (20 wt%), and sodium carboxymethyl cellulose (CMC, 10 wt%) in DI water, which were then applied to a copper foil using a doctor blade. A coin cell was fabricated in an argon-filled glove box and consisted of metallic potassium as the counter-electrode, porous polypropylene as the separator, and potassium bis(fluorosulfonyl) imide (KFSI, 1 M) dissolved in a mixture of ethylene carbonate/diethyl carbonate (EC/DEC, volumetric ratio of 1:1). The diameter of the electrode was 1.4 cm, and the mass loading of the electrode was 1.4 mg·cm^-2^. Galvanostatic charge/discharge and cyclic voltammetry (CV) measurements were carried out using a battery analyzer (WonATech, WBCS-3000s cycler) over the 0.001–3.0 V range of potentials, at various current densities. *In-situ* and *ex-situ* electrochemical impedance spectroscopy (EIS) measurements of the samples were performed, and the results were analyzed for frequencies ranging from 0.01 Hz to 100 kHz. During the in situ EIS analysis, the cell was cycled at a current density of 0.05 A g^-1^, and the samples’ Nyquist plots were obtained at preselected potentials.

**Supplementary Figures and Table**


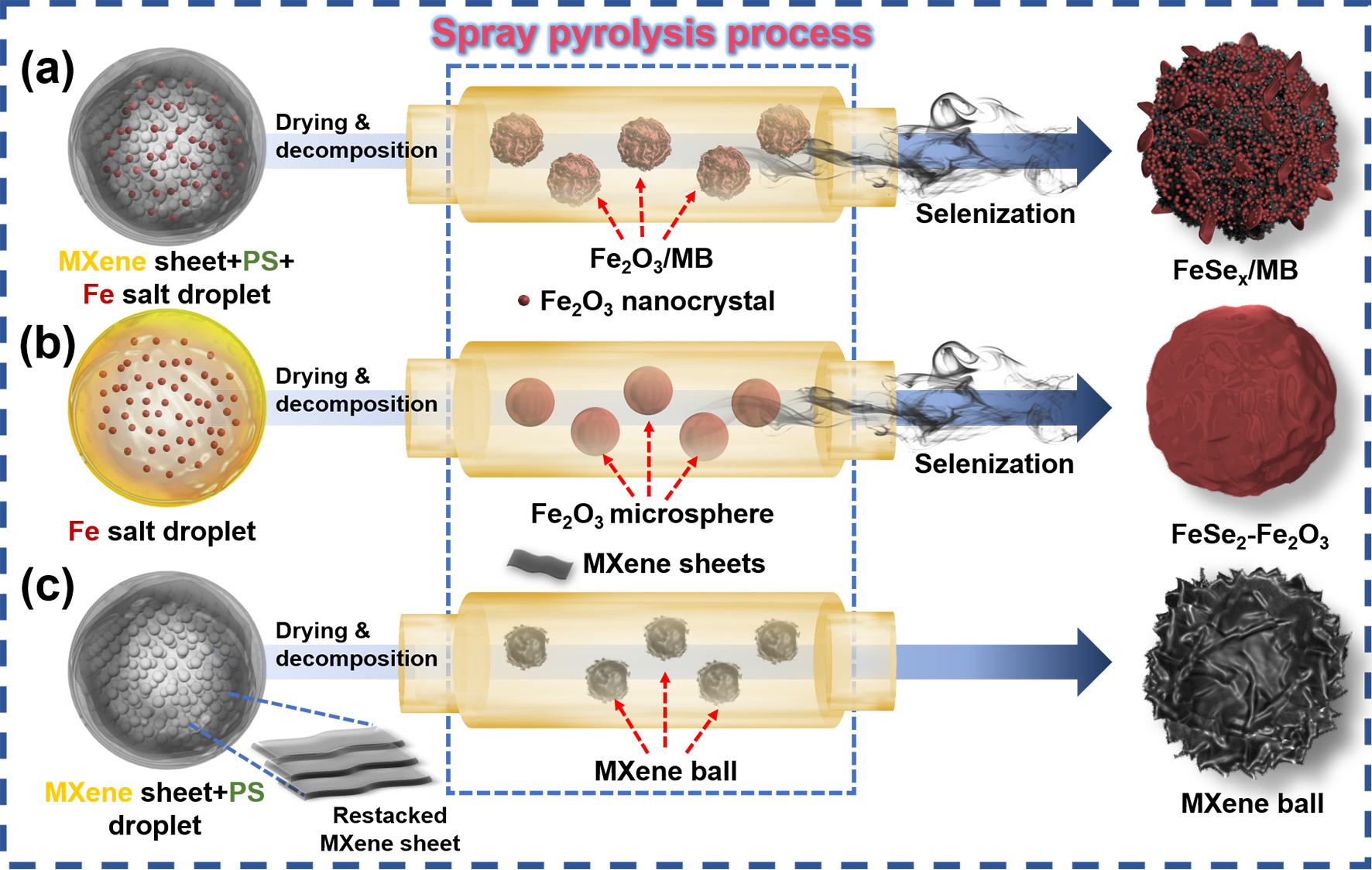


**Fig. S1** The formation mechanism of FeSe_x_/MB, bare FeSe_2_-Fe_2_O_3_, and MB


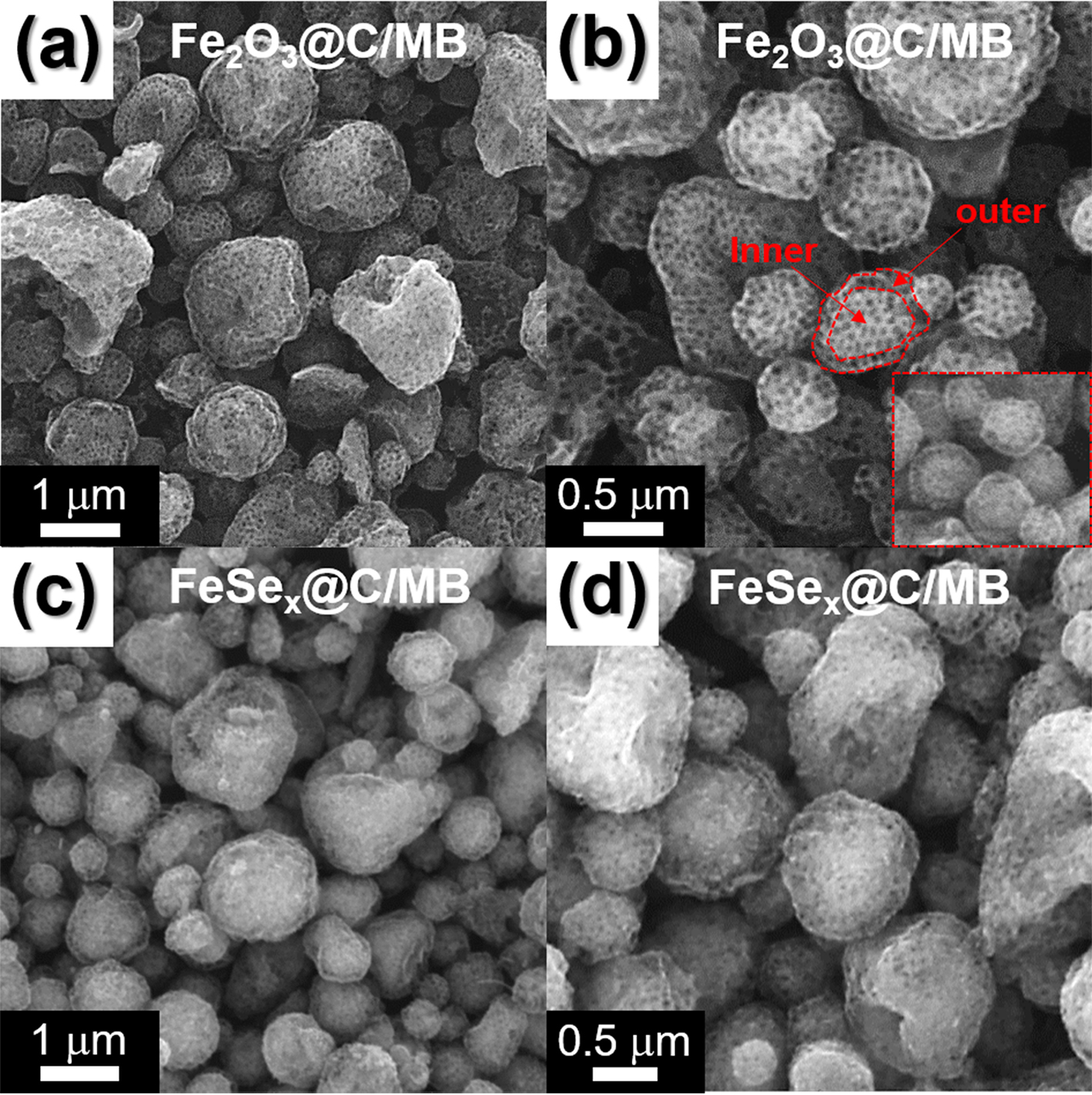


**Fig. S2** SEM images: **a, b** Fe_2_O_3_@C/MB, and **c, d** FeSe_x_@C/MB


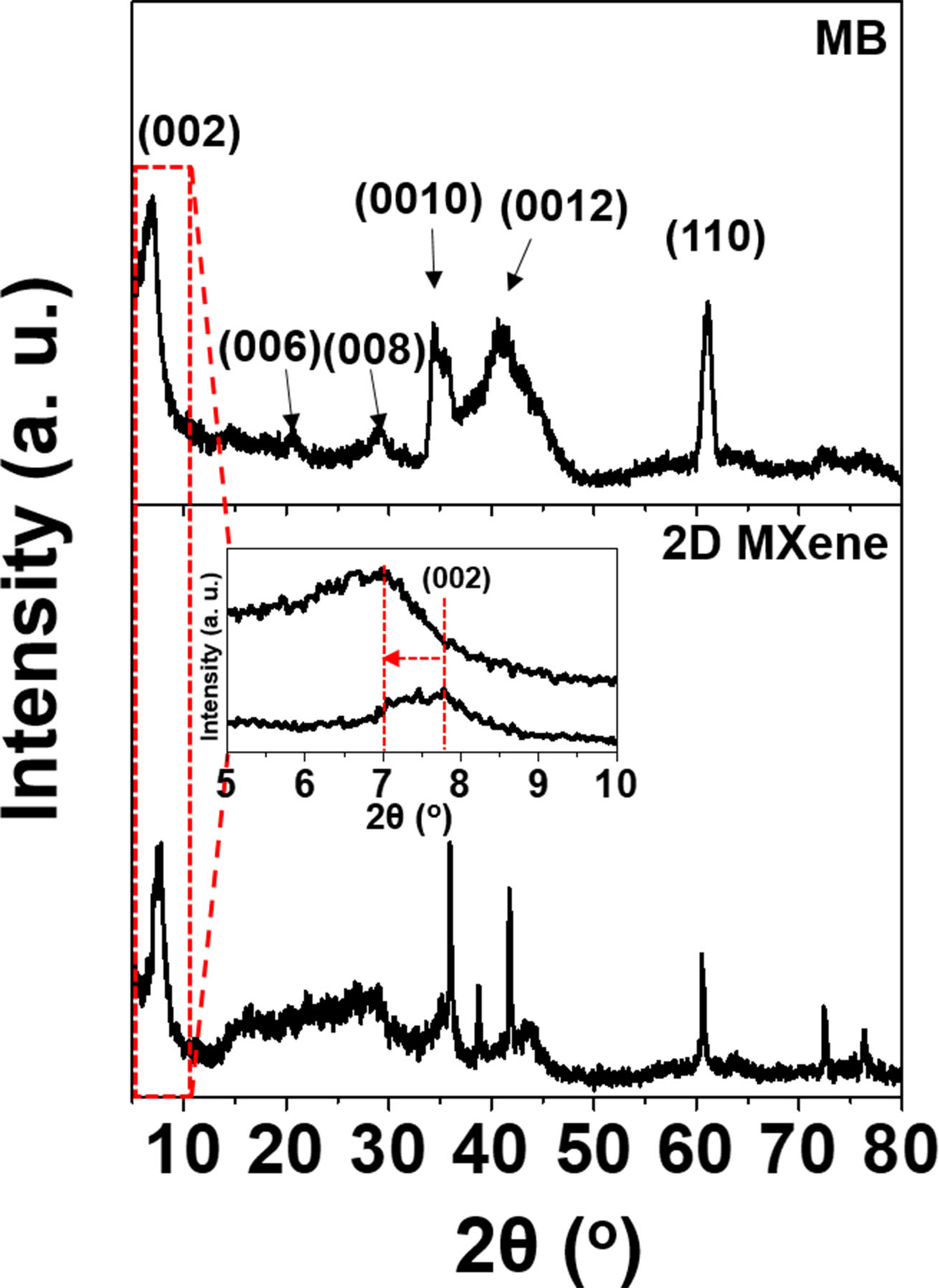


**Fig. S3** XRD patterns of MB and 2D MXene


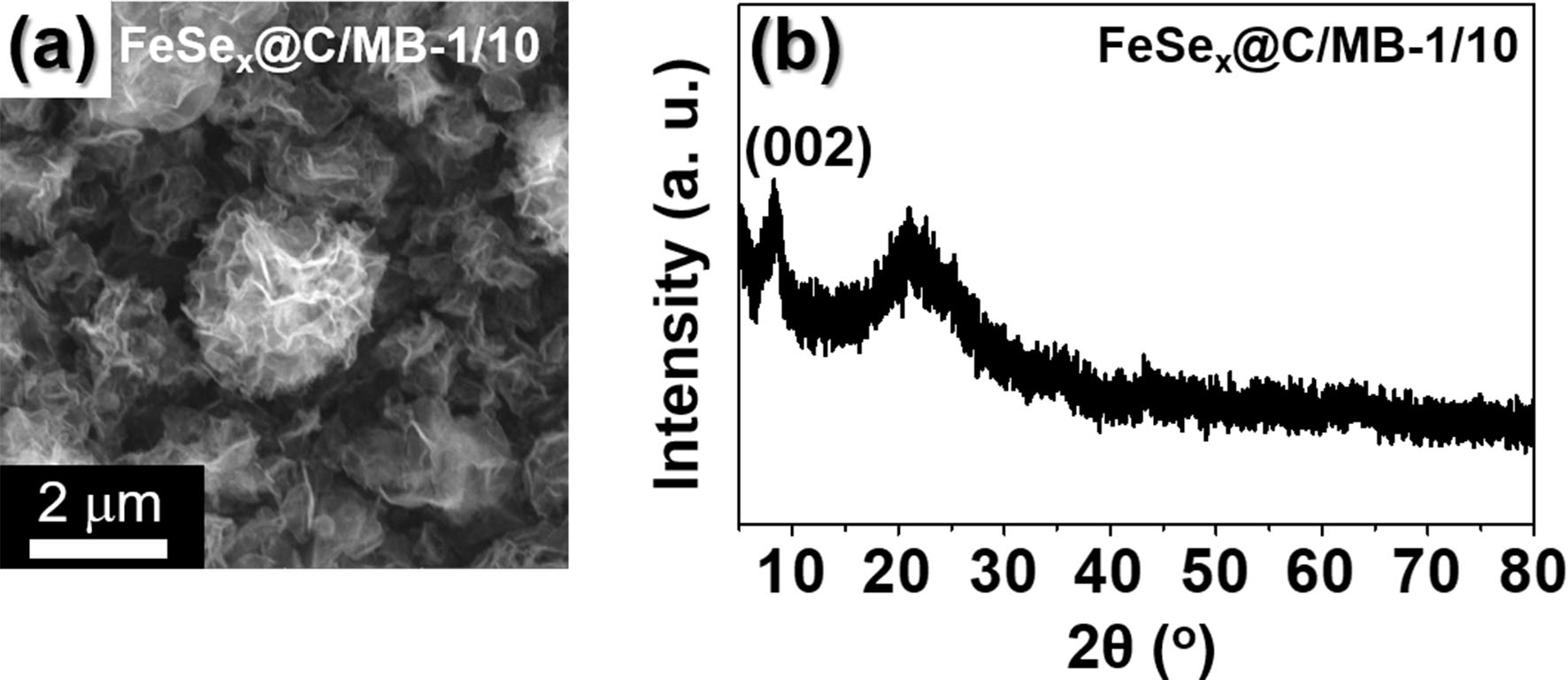


**Fig. S4 a** SEM image and **b** XRD data of 1/10 FeSe_x_@C/MB


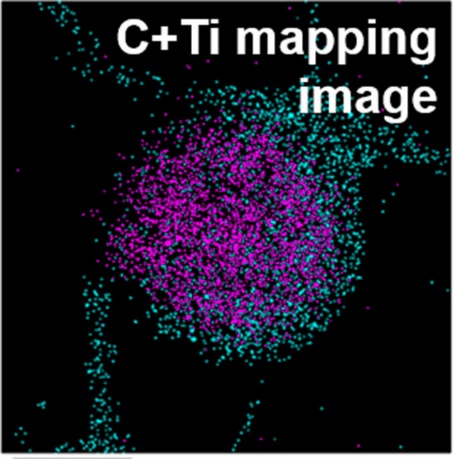


**Fig. S5** The distribution of C (blue color) and Ti (pink color) elements in FeSe_x_@C/MB


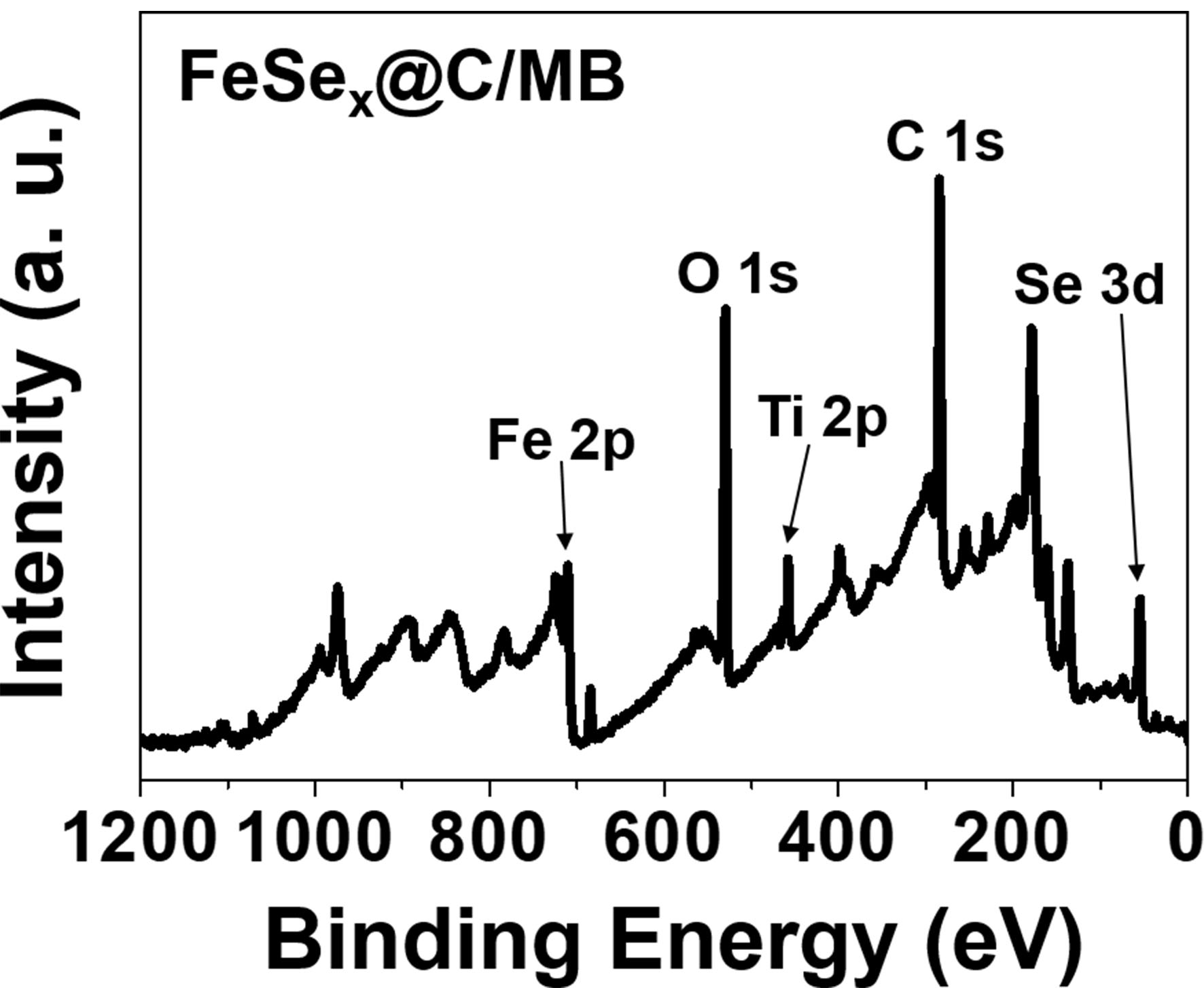


**Fig. S6** XPS survey scan for FeSe_x_@C/MB


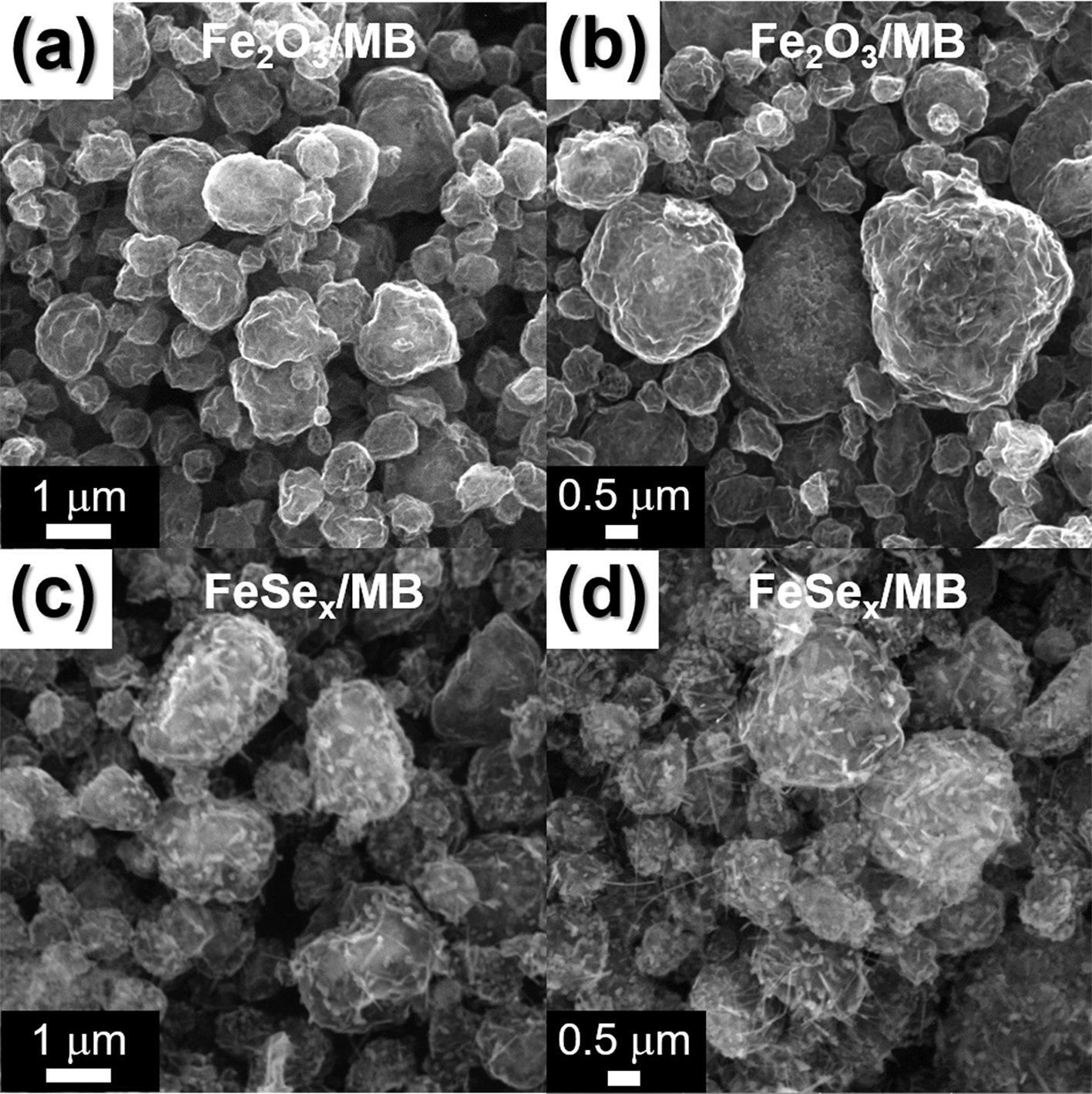


**Fig. S7** SEM images: **a, b** Fe_2_O_3_/MB, and **c, d** FeSe_x_/MB


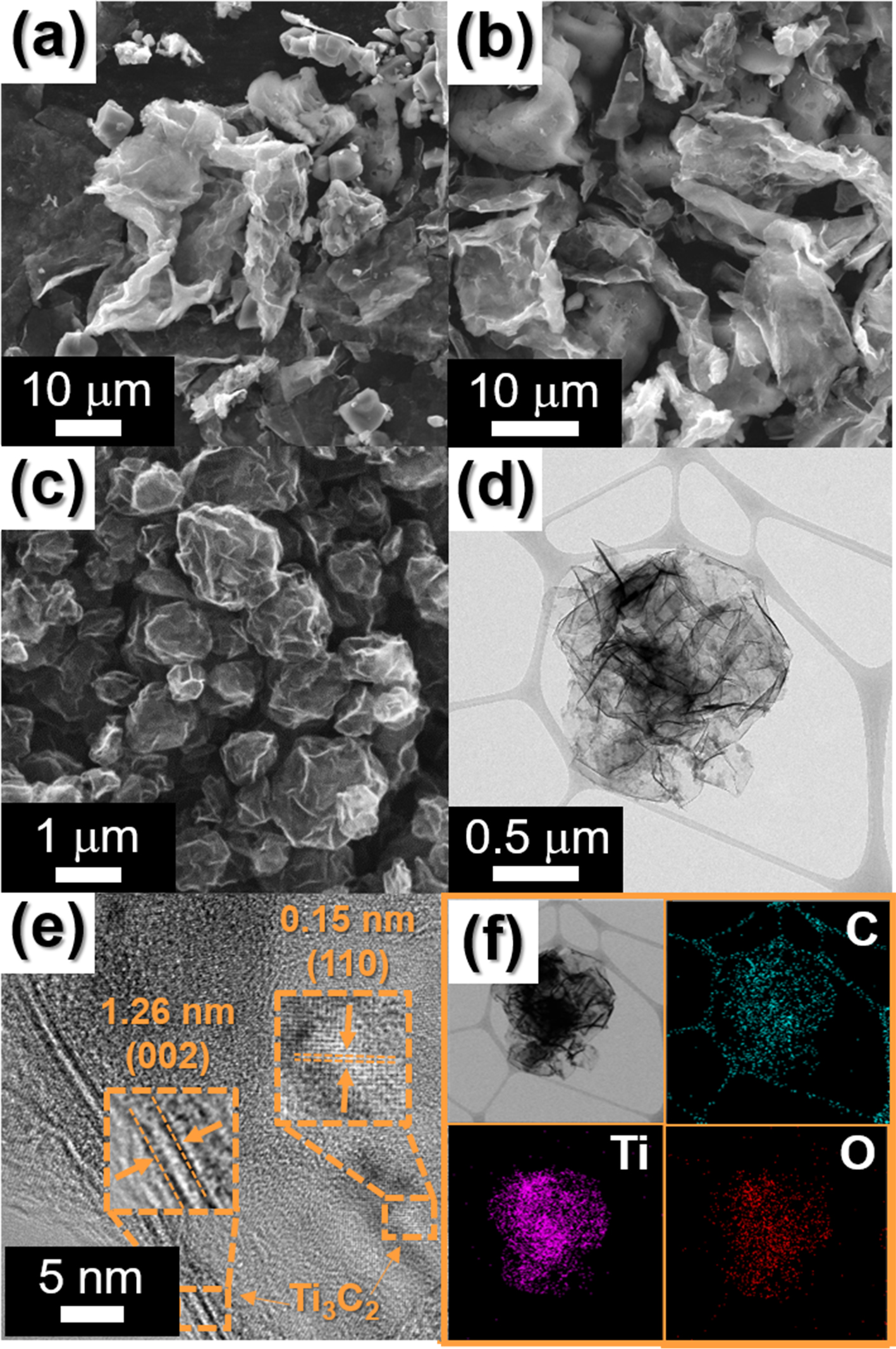


**Fig. S8** Morphologies, SAED, and elemental mapping images: **a, b** SEM images of 2D MXene nanosheets, **c** SEM image, **d** TEM image, **e** HR-TEM image, and **f** elemental mapping images of MB


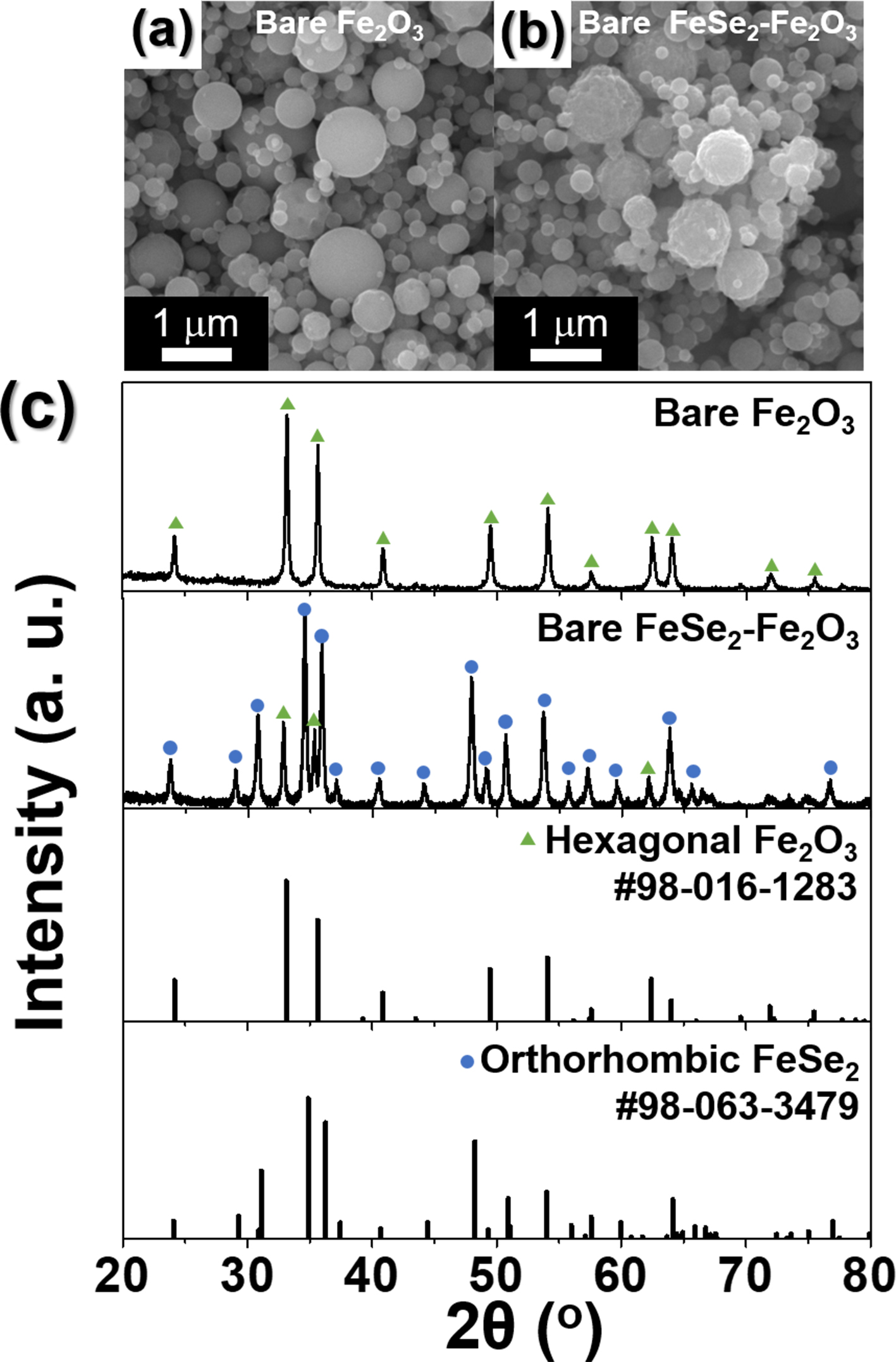


**Fig. S9 a, b** SEM images and **c** XRD data of bare Fe_2_O_3_ and FeSe_2_-Fe_2_O_3_


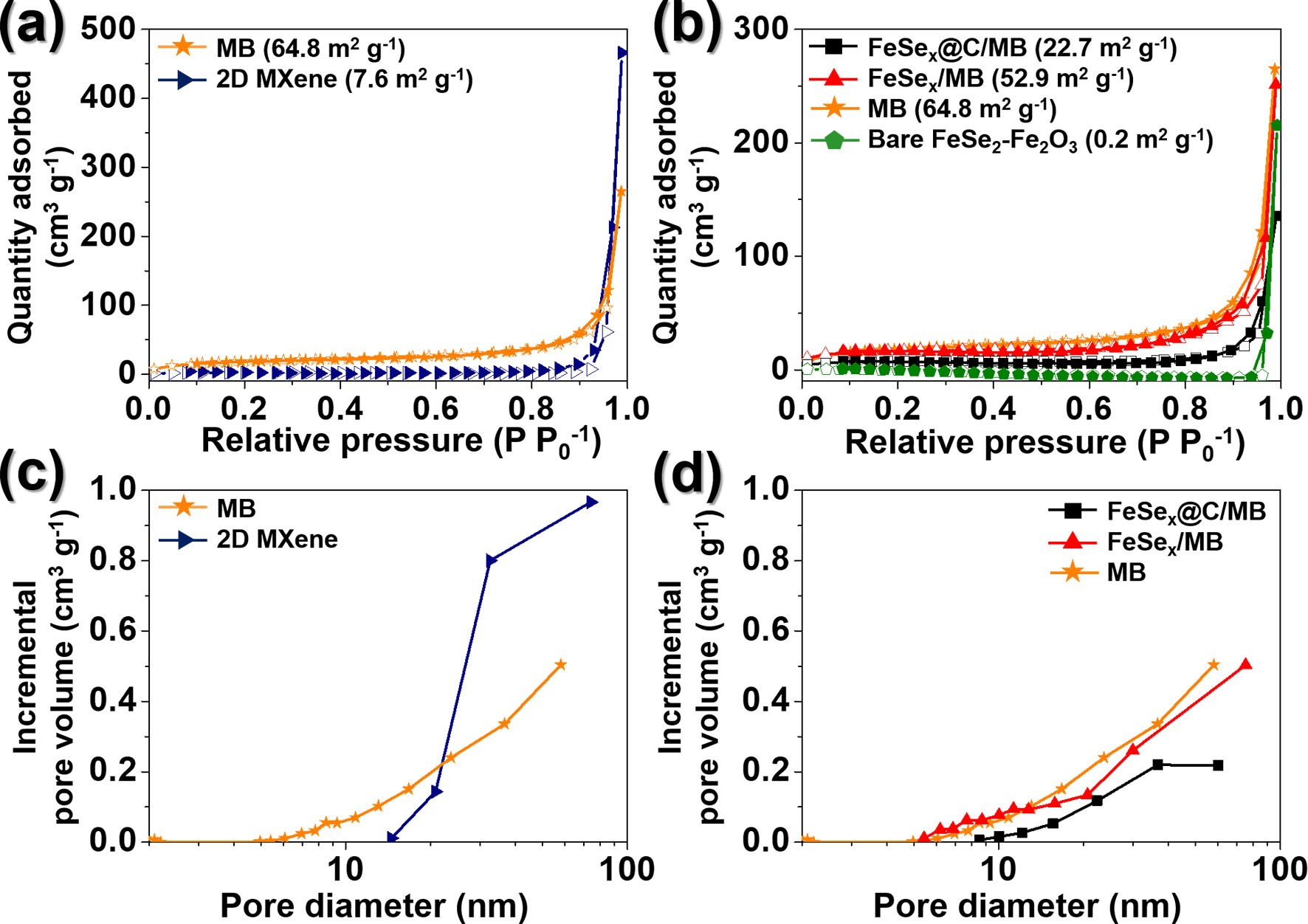


**Fig. S10 a, b** N_2_ gas adsorption and desorption isotherms, and **c, d** BJH pore size distributions of MB, 2D MXene, FeSe_x_@C/MB, FeSe_x_/MB, and bare FeSe_2_-Fe_2_O_3_


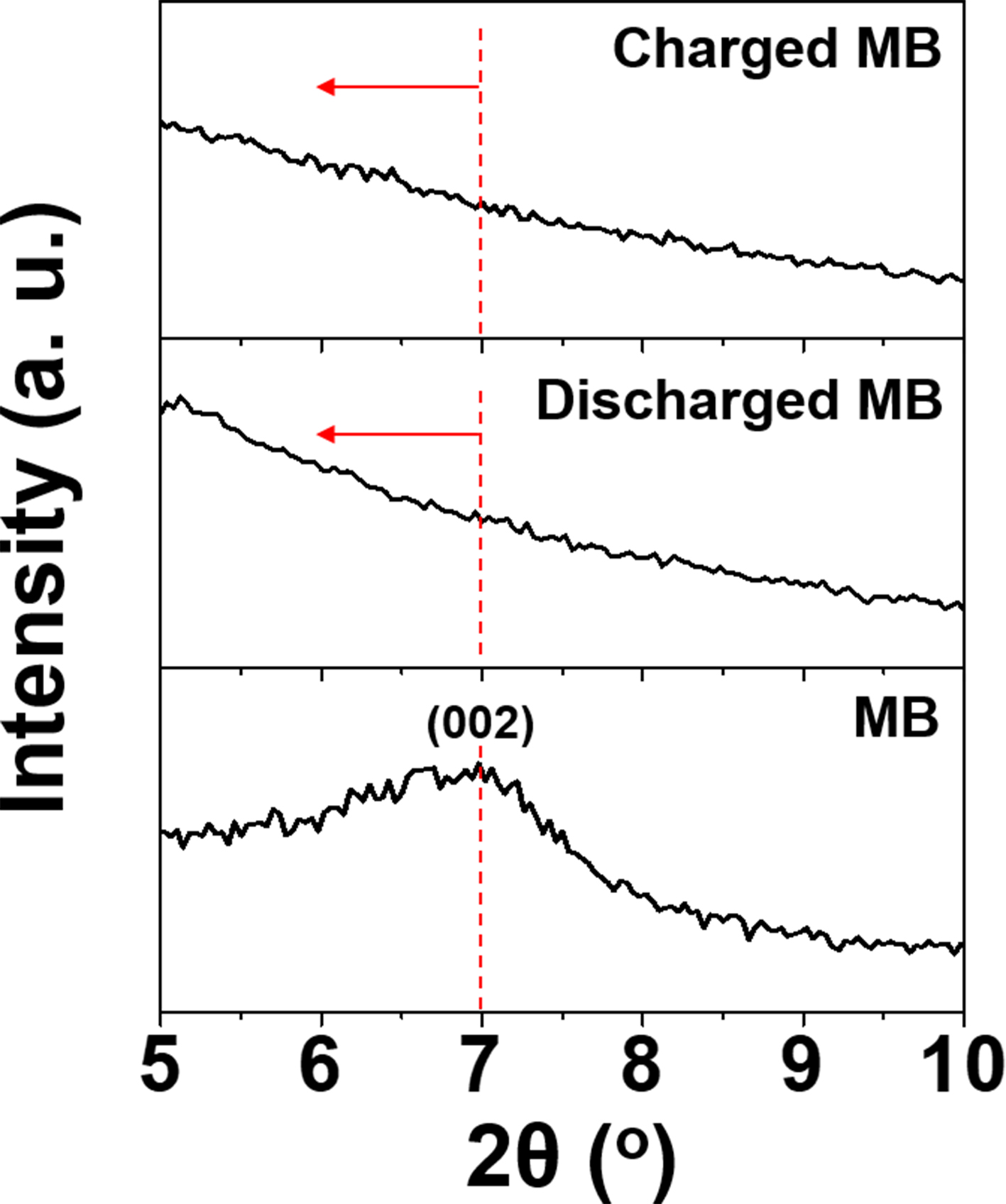


**Fig. S11** *Ex-situ* XRD pattern of MB after the first discharge, and charge state


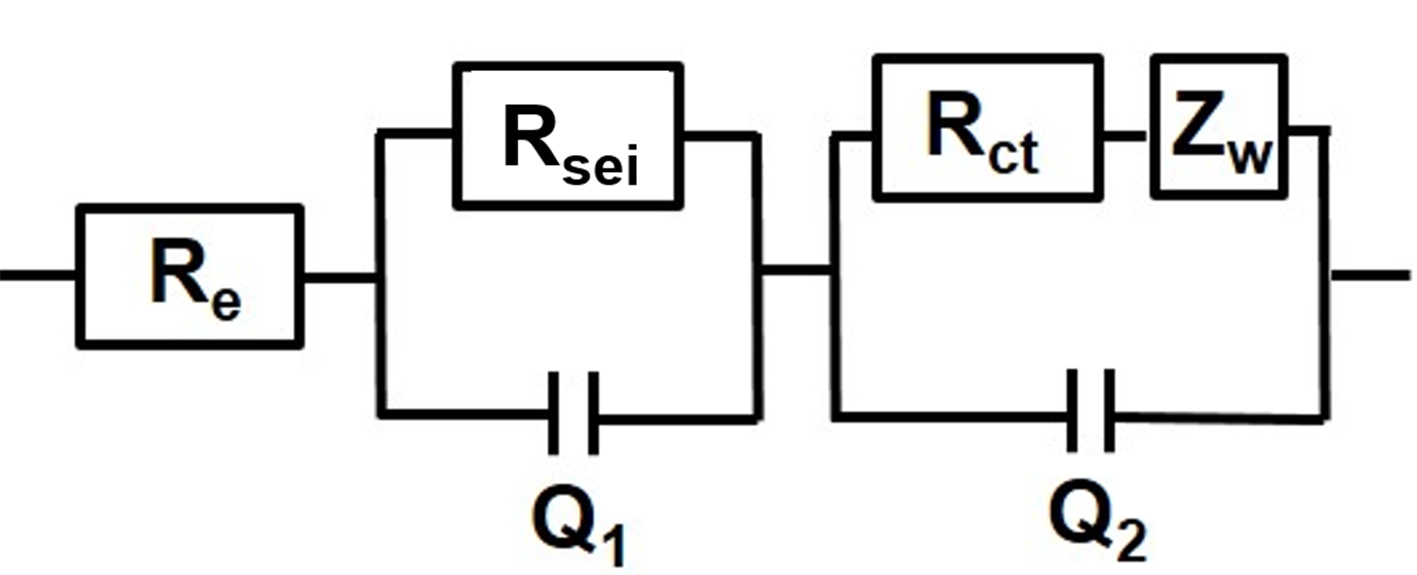


**Fig. S12** Randle-type equivalent circuit model used for EIS fitting

R_e_: Electrolyte resistance, corresponding to the intercept of high frequency semicircle at Z_re_ axis

R_sei_: SEI layer resistance corresponding to the high-frequency semicircle

Q_1_: Dielectric relaxation capacitance corresponding to the high-frequency semicircle

R_ct_: Charge transfer resistance related to the middle-frequency semicircle

Q_2_: Associated double-layer capacitance related to the middle-frequency semicircle

Z_w_: K-ion diffusion resistance


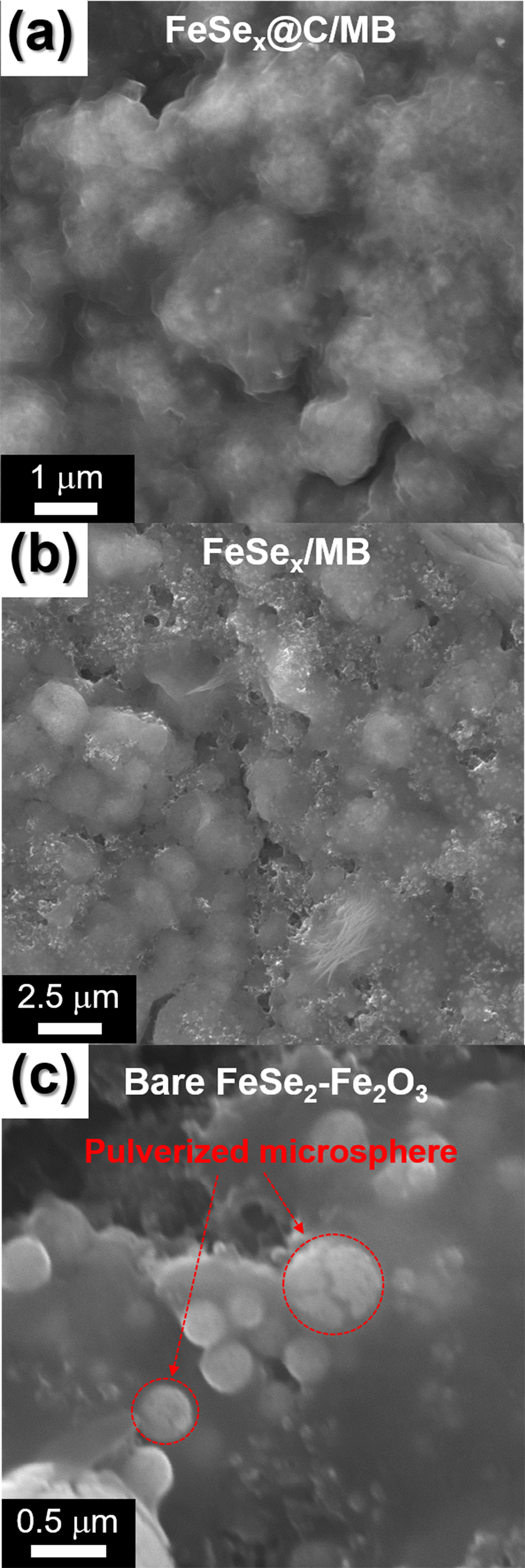


**Fig. S13** SEM images of **a** FeSe_x_@C/MB, **b** FeSe_x_/MB, and **c** bare FeSe_2_-Fe_2_O_3_ after 200 cycles.


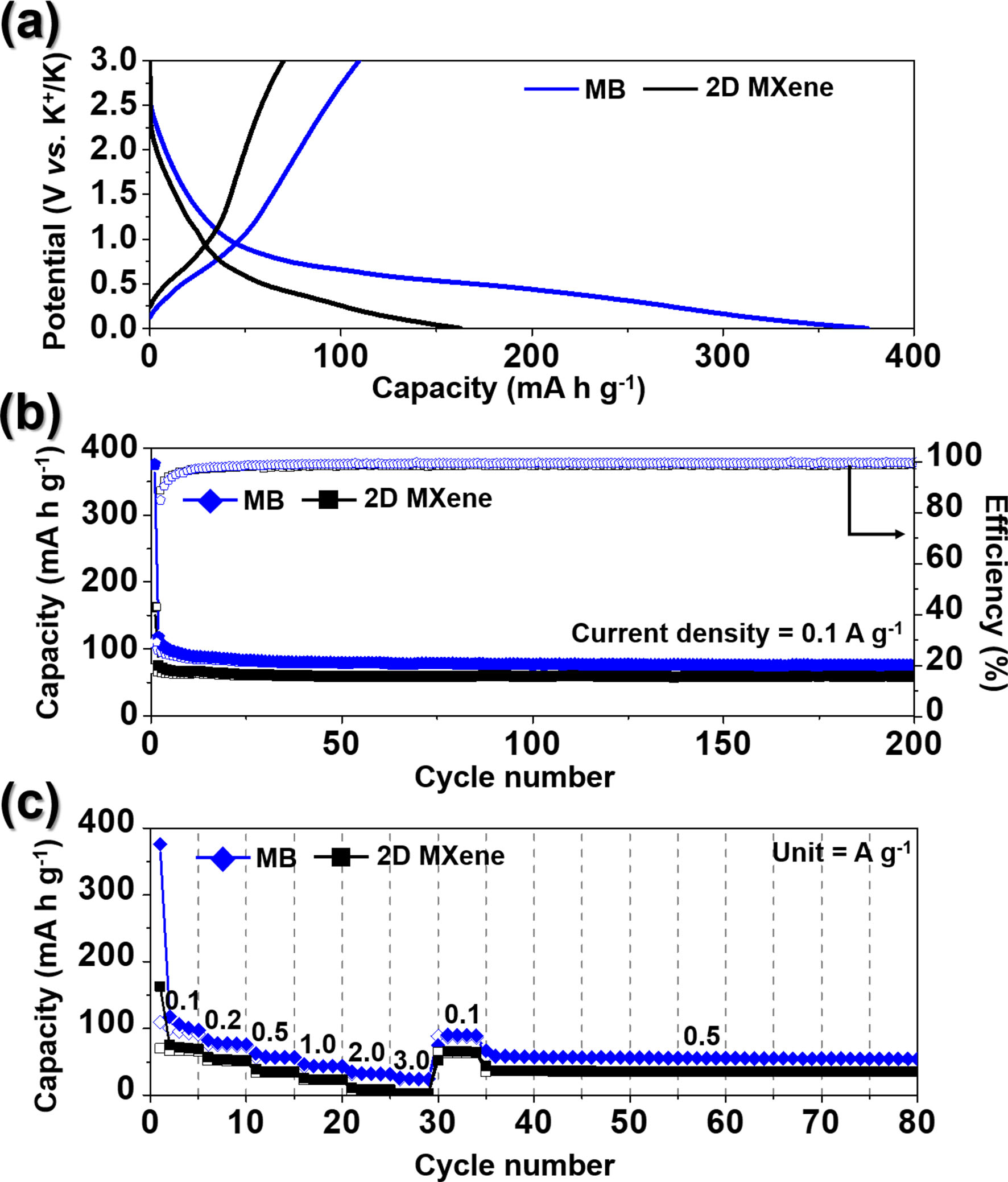


**Fig. S14** Electrochemical properties of MB and 2D MXene: **a** initial charge-discharge curves, **b** cycle performances at a current density 0.1 A g^-1^, and **c** rate performances at various current densities

**Table S1** Electrochemical properties of various nanostructured iron selenide anode materials applied as potassium-ion batteries reported in the previous literatures.

| **Materials** | **Voltage range (V)** | **Current rate** | **Discharge capacity**  **[mA h g^-1^] and (cycle number)** | **Rate capacity**  **[mA h g^-1^]**  **(current rate)** | **Ref** |
| --- | --- | --- | --- | --- | --- |
| **FeSe_x_@C/MB** | **0.001-3.0** | **0.1** | **410**  **(200)** | **169**  **(5.0 A g^-1^)** | **This work** |
| **FeSe_2_/NC** | 0.01-3.0 | 0.1 | 434  (70) | 341  (1.0 A g^-1^) | [S1] |
| **FeSe@C** | 0.01-3.0 | 0.5 | 298  (200) | 297  (1.0 A g^-1^) | [S2] |
| **Fe-Mo selenide@N-doped C** | 0.01-2.5 | 0.2 | 272  (100) | 227  (1.0 A g^-1^) | [S3] |
| **FeSe_2_@C** | 0.005-3.0 | 0.1 | 182  (100) | 61  (1.6 A g^-1^) | [S4] |
| **FeSe_2_@C NBs** | 0.7-3.0 | 0.1 | 221  (700) | 128  (1.0 A g^-1^) | [S5] |
| **Mn-Fe-Se/CNTs** | 0.0-3.0 | 0.05 | 141  (70) | 83  (0.8 A g^-1^) | [S6] |
| **FeSe_2_@C-3 MCs** | ~0.0-2.8 | 0.1 | 228  (100) | 142  (2.0 A g^-1^) | [S7] |
| **Fe_3_Se_4_@CF** | 0.01-2.0 | 0.05 | 357~  (50) | 77  (4.0 A g^-1^) | [S8] |

**References**

[S1] Y. Liu, C. Yang, Y. Li, F. Zheng, Y. Li, Q. Deng, W. Zhong, G. Wang, T. Liu. FeSe_2_/nitrogen-doped carbon as anode material for potassium-ion batteries. Chem. Eng. J. **393**, 124590 (2020). https://doi.org/10.1016/j.cej.2020.124590

[S2] J. Deng, X. Huang, W. Gao, H. Liu, M. Xu. 3D carbon framework-supported FeSe for high-performance potassium ion batteries. Sustain. Energy Fuels **4**, 4807-4813 (2020). https://doi.org/10.1039/D0SE00146E

[S3] J. Chu, Q. Yu, D. Yang, L. Xing, C.-Y. Lao, M. Wang, K. Han, Z. Liu, L. Zhang, W. Du, K. Xi, Y. Bao, W. Wang. Thickness-control of ultrathin bimetallic Fe–Mo selenide@N-doped carbon core/shell “nano-crisps” for high-performance potassium-ion batteries. Appl. Mater. Today **13**, 344-351 (2018). https://doi.org/10.1016/j.apmt.2018.10.004

[S4] T. Wang, W. Guo, G. Wang, H. Wang, J. Bai, B. Wang. Highly dispersed FeSe_2_ nanoparticles in porous carbon nanofibers as advanced anodes for sodium and potassium ion batteries. J. Alloys. Compd*.* **834**, 155265 (2020). https://doi.org/10.1016/j.jallcom.2020.155265

[S5] C. Liu, Y. Li, Y. Feng, S. Zhang, D. Lu, B. Huang, T. Peng, W. Sun. Engineering of yolk-shelled FeSe_2_@nitrogen-doped carbon as advanced cathode for potassium-ion batteries. Chin. Chem. Lett*.* (2021). https://doi.org/10.1016/j.cclet.2021.04.002

[S6] J. Wang, B. Wang, X. Liu, J. Bai, H. Wang, G. Wang. Prussian blue analogs (PBA) derived porous bimetal (Mn, Fe) selenide with carbon nanotubes as anode materials for sodium and potassium ion batteries. Chem. Eng. J*.* **382**, 123050 (2020). https://doi.org/10.1016/j.cej.2019.123050

[S7] S. Lu, H. Wu, S. Xu, Y. Wang, J. Zhao, Y. Li, A. M. Abdelkader, J. Li, W. Wang, K. Xi, Y. Guo, S. Ding, G. Gao, R. V. Kumar. Iron selenide microcapsules as universal conversion-typed anodes for alkali metal-ion batteries. Small **17**(8), 2005745 (2021). https://doi.org/10.1002/smll.202005745

[S8] A. Mahmood, Z. Ali, H. Tabassum, A. Akram, W. Aftab, R. Ali, M. W. Khan, S. Loomba, A. Alluqmani, M. Adil Riaz, M. Yousaf, N. Mahmood. Carbon fibers embedded with iron selenide (Fe_3_Se_4_) as anode for high-performance sodium and potassium ion batteries. Front. Chem. **8**, 408 (2020). https://doi.org/10.3389/fchem.2020.00408
